# Expression profiling of ALOG family genes during inflorescence development and abiotic stress responses in rice (*Oryza sativa* L.)

**DOI:** 10.3389/fgene.2024.1381690

**Published:** 2024-04-08

**Authors:** Zhiyuan Liu, Zhenjiang Fan, Lei Wang, Siyue Zhang, Weichen Xu, Sijie Zhao, Sijia Fang, Mei Liu, Sackitey Mark Kofi, Shuangxi Zhang, Ningning Kang, Hao Ai, Ruining Li, Tingting Feng, Shuya Wei, Heming Zhao

**Affiliations:** ^1^ Center for Crop Biotechnology, College of Agriculture, Anhui Science and Technology University, Fengyang, China; ^2^ College of Bioengineering, Wuhan Polytechnic University, Wuhan, China

**Keywords:** rice, ALOG family, expression analysis, inflorescence development, abiotic stress

## Abstract

The ALOG (*Arabidopsis* LSH1 and *Oryza* G1) family proteins, namely, DUF640 domain-containing proteins, have been reported to function as transcription factors in various plants. However, the understanding of the response and function of ALOG family genes during reproductive development and under abiotic stress is still largely limited. In this study, we comprehensively analyzed the structural characteristics of ALOG family proteins and their expression profiles during inflorescence development and under abiotic stress in rice. The results showed that OsG1/OsG1L1/2/3/4/5/6/7/8/9 all had four conserved helical structures and an inserted Zinc-Ribbon (ZnR), the other four proteins OsG1L10/11/12/13 lacked complete Helix-1 and Helix-2. In the ALOG gene promoters, there were abundant *cis*-acting elements, including ABA, MeJA, and drought-responsive elements. Most ALOG genes show a decrease in expression levels within 24 h under ABA and drought treatments, while *OsG1L2* expression levels show an upregulated trend under ABA and drought treatments. The expression analysis at different stages of inflorescence development indicated that *OsG1L1*/*2*/*3*/*8*/*11* were mainly expressed in the P1 stage; in the P4 stage, *OsG1*/*OsG1L4*/*5*/*9*/*12* had a higher expression level. These results lay a good foundation for further studying the expression of rice ALOG family genes under abiotic stresses, and provide important experimental support for their functional research.

## 1 Introduction

Rice (*Oryza sativa*) is one of the most important food crops in the world and an ideal model plant for genomics research and monocotyledonous plant evolutionary lineage research (Chen et al., 2022). Some studies had reported that ALOG genes involved in the regulation of lemma, palea and inflorescence development in rice. For example, *OsG1* inhibits the homologous transformation of sterile lemma to normal lemma, and *TH1*/*BH1*/*AFD1* affects the development of rice lemma/palea, spikelet morphogenesis, and grain shape. It has been reported that both genes belong to the ALOG family (Li et al., 2019). The characteristic of members of the *Arabidopsis* LSH1 and *Oryza* G1 (ALOG) gene family is the conserved DUF640 domain which is considered specific transcription factor in plants (Yoshida et al., 2009; Chen et al., 2019). The ALOG domain is suggested to originate from the N-terminal DNA-binding domains of the XerC/D-like tyrosine recombinases encoded by a novel category of DIRS1-like LTR (long terminal repeat) retrotransposon found in several eukaryotes. The N-terminus of the ALOG domain fused with a specific but weakly catalytic N6-adenine methylase active region, while the C-terminus fused with a tyrosine recombinase catalytic active region. These two regions are prominent features of DIRS1-like retrotransposons in eukaryote (Poulter and Goodwin, 2005). The secondary structure predictions showed that the ALOG domain consisted of four conserved α-helices, and an additional Zinc-Ribbon (ZnR) was inserted between helix-2 and helix-3 with “HxxxC” and “CxC” motifs, making this domain more flexible and facilitating binding to more DNA (Iyer and Aravind, 2012).

Current researches indicate that the ALOG genes play an important role in seedling growth, floral organ development and inflorescence meristem differentiation (Li et al., 2019). *LIGHT-DEPENDENT SHORT HYPOCOTYLS 1* (*LSH1*) is the first ALOG gene identified from *Arabidopsis*, and mainly expressed in hypocotyls, shoot tips, and lateral root primordia. The functional analysis suggested that *AtLSH1* might play a role as a transcriptional regulatory protein to control seedling development in light-dependent manner (Zhao et al., 2004). Overexpression of *AtLSH1* and *AtLSH2* significantly inhibit the hypocotyls elongation and reproductive growth of *Arabidopsis* (Lee et al., 2020). *LSH4* and its homologous gene *LSH3* are expressed in the boundary cells of various bud organs, and inhibit bud organ differentiation in boundary region during plant development (Takeda et al., 2011). Moreover, the expression of *LSH4* in the shoot apex inhibits leaf growth during the vegetative period and the formation of additional shoot organs within the flower during the reproductive period. *LSH8* participates as a positive regulatory factor in the ABA signaling pathway during seed germination and seedling growth in *Arabidopsis* (Zou et al., 2021). *LSH9* regulates hypocotyl elongation by interacting with temperature receptor *ELF3* (Press and Queitsch, 2017). *LSH10* is found to interacts with *OTLD1* in plant cells to form an *OTLD1-LSH10* co-repressor protein complex that deubiquitinates histone 2B and inhibits the expression of genes involved in growth, cell expansion, and hormone signaling (Vo Phan et al., 2023). These researches indicated that the ALOG genes in *Arabidopsis* were involved in vegetative and reproductive development.

*OsG1*, the first gene reported in the rice ALOG family, is mainly expressed in the sterile lemma primordia during their development, and the sterile lemma in the *g1* mutant is larger than WT. The further analysis reveals that *OsG1* may serve as a transcription inhibitor and specify the identity of sterile lemma by regulating downstream hormone signal transduction (Yoshida et al., 2009). Similarly, *OsG1L6* regulates the development of rice lemma and palea by controlling cell division and expansion, thereby affecting grain shape and size (Yan et al., 2013). In addition, *OsG1L6* acts as a transcription inhibitor and regulates cell expansion during lateral development of spikelets (Ren et al., 2016; Peng et al., 2017). *OsG1L5*, as known as *TAW1* (*TAWAWA1*), is highly expressed in shoot apical meristem, and regulates inflorescence development by maintaining inflorescence meristem (IM) activity and inhibiting the phase transitions of spikelet meristem (SM) identity (Yoshida et al., 2013). Similar to *OsG1L5*, *OsG1L1* and *OsG1L2* are also expressed at high levels in the IM tissues, and regulate inflorescence branching in rice. Compared with the WT, *osg1l1* and *osg1l2* had shorter panicles, fewer spikelets, and smaller grains, farther the inflorescence structure of the mutants’ phenotype are similar to that of the *TAW1* (Beretta et al., 2023). *TtTAW1-1A*, the homologous gene of *OsG1L5* in *Triticum turgidum* participates in regulating the development of branching and meridians in wheat (Nan et al., 2018). In *sorghum*, *DOMINANT AWN INHIBITOR (DAI)*, derived from gene replication, encodes an ALOG protein, which may inhibit awn elongation by acting as a transcription factor to repress cell proliferation and elongation in *sorghum* (Takanashi et al., 2022). Overexpression of *PhLSH7a* and *PhLSH7b* in *Arabidopsis* resulted in rounded leaves, late flowers and deformed flowers, indicating that these two genes significantly affected the vegetative and reproductive growth (Chen et al., 2019). An ALOG protein in *Solanum lycopersicum*, Terminating Flower (TMF), affects panicle architecture by inhibiting inflorescence meristem tissue (MacAlister et al., 2012). These research results indicated that the ALOG family genes involved in regulation of the reproductive and developmental processes of plants, but its function under abiotic stress was still unclear.

Improving rice yield and tolerance to abiotic stress had always been a research hotspot (Reyes, 2023), and some ALOG family members had been reported to be essential for rice grain development. However, the analysis of ALOG family members was not systematic enough. Therefore, this study conducted amino acid sequence alignment, 3D protein structure prediction, phylogenetic analysis, collinearity analysis, promoter *cis*-acting element analysis, expression analysis during inflorescence development and under abiotic stresses of the ALOG family in rice. This study is crucial for understanding the evolution and diversity of rice ALOG genes and their potential roles in plant growth and stress response.

## 2 Materials and methods

### 2.1 Plant materials and growth conditions

In order to analyze the expression profile of the *ALOG* genes during vegetative and reproductive development, the rice plants of *O. sativa* japonica cv. Zhonghua 11 (ZH11) were grown under normal field conditions in summer at Anhui Science and Technology University. The different tissues were used, including 7-day-old seedlings (YS), 80-day-old roots (R), 80-day-old stems (St), 80-day-old leaves (YL), seeds at 3 DAP (day after pollination, S2) and at 5 DAP (S3), different stages of panicle development: P1, 0–1 cm; P2, 1–3 cm; P3, 3–5 cm; P4, 5–10 cm; P5, 10–15 cm; P6, 15–22 cm.

For expression analysis of ALOG genes in response to ABA and drought treatments, the rice seeds of ZH11 were germinated and sown in the 96 well boxes with nutrient solution in the greenhouse at 25°C–30°C and 60% humidity with a photoperiod of 16 h/8 h (light/dark). The 14-day-old seedlings were placed in nutrient solution containing 200 μM ABA or 20% PEG6000, respectively. The samples were selected at 0, 3, 6, 12, and 24 h after the treatments. Parallel control samples were prepared by keeping the seedlings in nutrient solution. All materials contained three biological replicates, and were immediately frozen in liquid nitrogen and stored at −80°C for RNA extraction.

### 2.2 Multiple sequence alignments and prediction of 3D protein structure

For multiple sequence alignments, the full-length protein sequences of the ALOG family members in rice and *Arabidopsis* were obtained from the NCBI (https://www.ncbi.nlm.nih.gov) and TAIR (https://www.arabidopsis.org) databases, respectively (Supplementary Table S1). The MUSCLE program of MEGA 11 was used to align amino acid sequences of the ALOG domain (about 134 aa) in rice and *Arabidopsis*. Then, the GeneDoc software was used to color the regions with different degrees of sequence conservation.

The initial 3D protein structure was performed by using the AlphaFold2 program based on amino acid sequences of the ALOG family in rice, and the ALOG domains of the protein 3D structures were colored in the PyMOL software (https://pymol.org/2/).

### 2.3 Phylogenetic analysis of ALOG

The ALOG family protein sequences in *Brachypodium distachyon*, *Sorghum bicolor*, and *S. lycopersicum* were obtained from the online database phytozome 13 (https://phytozome-next.jgi.doe.gov/blast-search) (Supplementary Table S1). MEGA 11 was used to align the protein sequences of the ALOG families with the ClustalW program with parameters set to default values. Using the method of Maximum Likelihood, a phylogenetic tree was constructed for the protein sequences of ALOG family members, with a bootstrap repeat value of 1,000, and other parameters were defaulted. The phylogenetic trees were visualized using the Interactive Tree of Life (iTOL) program.

### 2.4 Gene structure, motif analysis and collinearity analysis

The coding sequences (CDS) (Supplementary Table S2) and genomic sequences (Tbale S3) of the *OsG1/OsG1L1-13* were used to analyze the exon-intron structures in the GSDS (Gene Structure Display Server 2.0) website (gsds.gao-lab.org).

The rice ALOG family protein sequences were submitted to the MEME suite (http://meme-suite.org/tools/meme) and processed with a maximum discovery number of motifs set to 10, and then the result file was visualized through the TBtools.

The GFF3 annotation files of rice, *Arabidopsis* and *B. distachyon* were sourced from the database ensemblplants (http://plants.ensembl.org/info/data/ftp/index.html). Based on the GFF3 files, the MCScanX program was used to analyze the repeated events of ALOG genes (Wang et al., 2012). The conserved synteny between rice and other two species were analyzed using the Dual Synteny Plotter program in TBtools.

### 2.5 Subcellular localization analysis of OsG1L3 and OsG1L7

In order to verify the subcellular localization prediction of ALOG members, the CDS sequences of *OsG1L3* and *OsG1L7* were amplified from ZH11 cDNA using the specific primers (Supplementary Table S4), and fused with vector *pCAMBIA2300*. The recombinant constructs (35S::OsG1L3/7-GFP) and the empty construct (35S::GFP) were transformed separately into rice protoplasts. The GFP signals were observed by a laser scanning microscope (C2-ER, Nikon, Japan) after culture for 16 h, and the excitation and emission wavelength was 488 and 510 nm, respectively.

### 2.6 Expression data analysis of ALOG genes in rice

The microarray data of ALOG genes in rice were extracted from the Rice Functional Genomic Express Database (http://signal.salk.edu/cgi-bin/RiceGE), and then were used to analyze expression profiles of the genes in the tissues during different development stages (GSE6893, GSE6901, GSE7951). The absolute signal values in the microarray data were respectively divided by the average of all absolute values, and the logarithmic values of the ratios were used to generate hierarchical cluster display using the Heatmap function in software TBtools.

### 2.7 *Cis*-regulatory elements analysis of ALOG genes in rice

To understand the *cis*-regulatory elements in the promoter region of ALOG genes, we used TBtools to retrieve the genomic sequences of 3,000 bp upstream of the start codon (ATG), and then submitted them to PlantCARE online website (http://bioinformatics.psb.ugent.be/webtools/plantcare/html) to predict the *cis*-acting elements, notably ABA and drought stress response elements. The results were visualized using Simple Biosequence Viewer and HeatMap program in the TBtools.

### 2.8 RNA isolation and qRT -PCR analysis

The extraction and quality detection of total RNA from all collected samples were carried out according to the method of Gan et al. (2022). For quantitative real time PCR, 1 μg of RNA was reverse transcribed into cDNA using the MonScriptTM RTIII AII-in-One Mix with dsDNase (Monad, Wuhan) kit according to the instruction manual, and qRT-PCR was performed with the SYBR Green Ⅰ PCR Master Mix system on the ABI ViiA7 real-time fluorescence quantitative PCR instrument (Life Technologies, USA). The gene-specific primers of *OsG1*/*L*s were listed in Supplementary Table S5. The reaction system is 20 μL: 10 μL for ProQTM qPCR EvaGreen Master Mix (Biomed), 2 μL cDNA, 0.5 μL Forward primer and 0.5 μL Reverse primer, ddH_2_O up to 20 μL. The qRT-PCR program was: 95°C for 2 min; 40 cycles of denaturation at 95°C for 15 s, annealing at 60°C for 15 s and extension at 72°C for 30 s. The *Actin* gene was used as the internal control in rice. The relative expression levels of the genes examined were evaluated using the 2^−ΔCT^ method (Livak and Schmittgen, 2001). The mean values ±SD (standard deviation) were calculated in the SPSS 26.0 software (*p* < 0.05), and the software GraphPad Prism 8.0 was used to visualize data.

## 3 Results

### 3.1 Multiple sequence alignment and protein three-dimensional structure prediction of ALOG proteins

**FIGURE 1:**
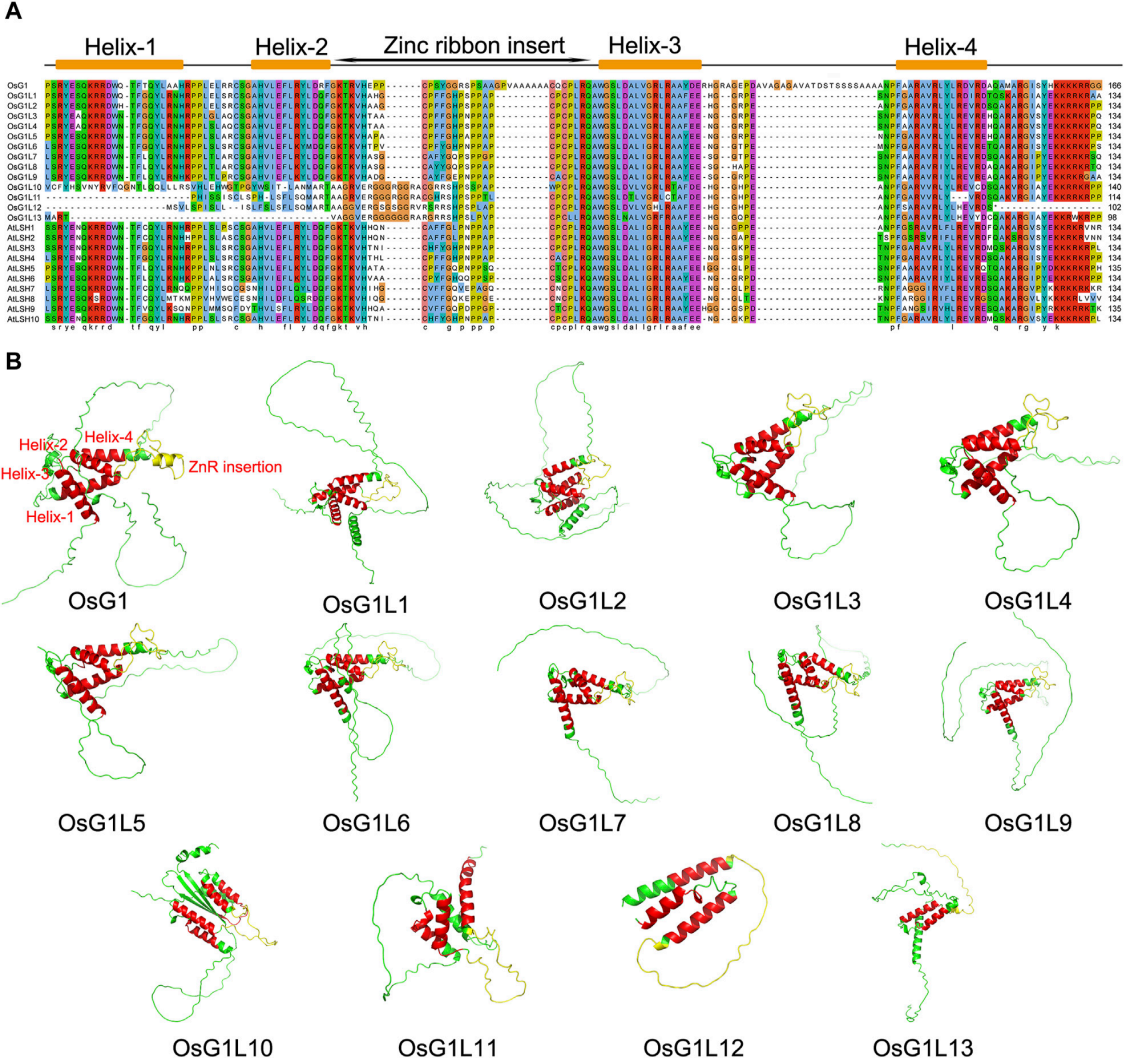
Multiple sequence alignment and 3D analysis of ALOG proteins. **(A)** Multiple sequence alignment of the ALOG proteins from rice and *Arabidopsis thaliana*. Multiple alignments of the highly conserved ALOG domains (134 aa) of ALOG proteins. The conserved ALOG domain included Helix-1/2/3/4 and a zinc ribbon insert structure. **(B)** Prediction of three-dimensional structure of 14 ALOG proteins from rice. Helix-1, Helix-2, Helix-3 and Helix-4 were marked in red, and the zinc ribbon insert was marked in yellow.

**FIGURE 2:**
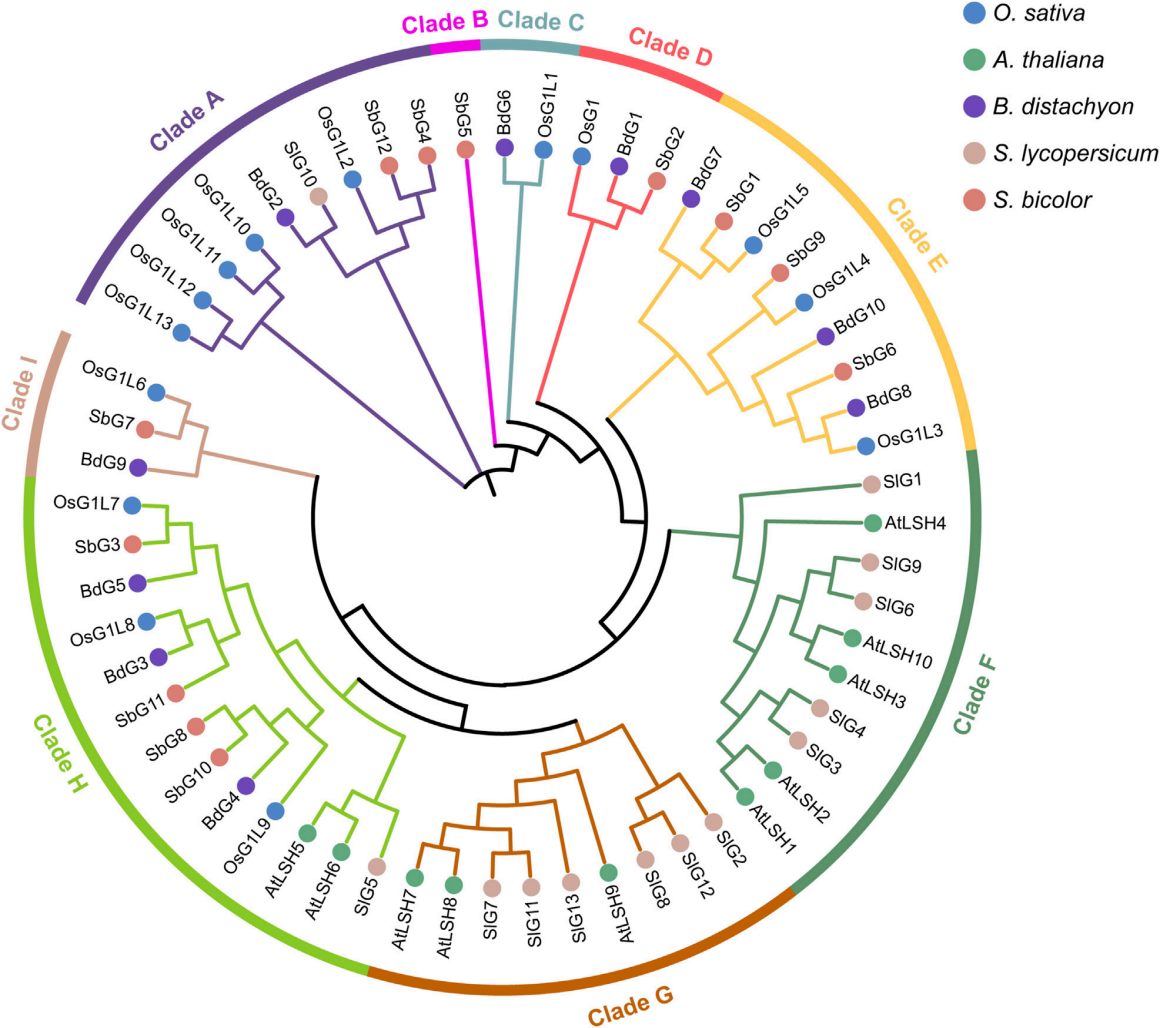
Phylogenetic tree of ALOG domain proteins in five different plant species. On the basis of the full-length sequences of ALOG proteins, a total of 59 proteins were used to construct the phylogenetic tree. Species abbreviations were as follows: Os, *Oryza sativa*; At: *Arabidopsis thaliana*; Bd: *Brachypodium distachyon*; Sl: *Solanum lycopersicum*; Sb: *Sorghum bicolor*. Clades A-I were indicated in different colored circles.

According to previous reports, there were 14 members in the rice ALOG family (Li et al., 2019), and we collected basic information of 14 ALOG members (Supplementary Table S6). To better understand the sequence characteristics of ALOG domain, the sequences of ALOG proteins from rice and *Arabidopsis* were aligned and analyzed (Figure 1A). The results showed that most of ALOG proteins contained a conserved domain with 134 aa, which consisted of Helix-1/2/3/4 and a zinc ribbon inserted between Helix-2 and Helix-3. However, 10 out of the 14 ALOG proteins from rice included all conserved domain, of which OsG1L10 had a partial change in its first helical structure Helix-1, OsG1L11/12 lost the Helix-1, and OsG1L13 lost Helix-1 and Helix-2, and part of Helix-4 were only missing in OsG1L12 (Figure 2A). These results were consistent with previous reports (Iyer and Aravind, 2012), and showed that most ALOG proteins were highly conserved.

Protein conformation is usually related to their function (Varadi et al., 2022). To further explore the conservation and difference of ALOG domains, the three-dimensional (3D) structures of 14 ALOG proteins from rice were predicted through Alphafold2 program (Jumper et al., 2021). The results showed that the secondary structure of all ALOG proteins contained a zinc ribbon insert, and four helix structures existed in OsG1/OsG1L1/2/3/4/5/6/7/8/9/10 (Figure 1B). Among the other three proteins, OsG1L11/12/13, Helix-1 were absent in OsG1L11 and OsG1L12. Both of Helix-1 and Helix-2 were missing in OsG1L13. Moreover, it was noteworthy that four helix structures were parallel to each other in OsG1L10, which was different from the helix structures of conformation in other proteins.

### 3.2 Phylogenetic, gene structure and protein motif analysis of ALOG members

To explore the evolutionary relationship and functional differences among ALOG members, the phylogenetic tree was constructed using the full-length sequences of ALOG proteins from rice and other four species, including *Arabidopsis thaliana*, *B. distachyon*, *S. lycopersicum*, and *Sorghum bicolor* (Figure 2). On the basis of phylogenetic analysis, 59 ALOG proteins from five species were divided into nine different clades (Clade A-I) in the phylogenetic tree. Among nine clades, the ALOG proteins in clades A, B, C, D, E and I were all from monocotyledons, the ALOG proteins in clade F and G were only from dicotyledons. Differently, the clade H contained not only three proteins from dicotyledons but also 10 proteins from monocotyledons. Particularly, the clade B was only composed of SbG5. 14 ALOG proteins from rice were unevenly distributed on six clades, clade A containing OsG1L2/10/11/12/13, clade C including OsG1L1, clade D including OsG1, clade E including OsG1L3/4/5, clade H containing OsG1L7/8/9 and clade I including OsG1L6.

To further understand the sequence conservation and divergence of rice ALOG members, gene structrue and conserved motifs were identified in the 14 ALOG proteins. Gene structure analysis revealed that half of rice ALOG genes (*OsG1L2*/*3*/*4*/*5*/*6*/*10*/13) contained introns, of which *OsG1L2*/*5*/*10* had only one intron and *OsG1L3*/*4*/*6*/*13* had two introns. The members of the same clade in the phylogenetic tree had similar exon/intron structures, with *OsG1L7*/*8*/*9* (belong to clade H) not containing introns and *OsG1L3*/*4* containing two introns (Supplementary Figure S1A, B). Motif analysis showed that a total of 10 motifs were present in ALOG proteins and the number of motifs in each protein varies from five to eight (Supplementary Figure S1C). Motifs one to five constituted the ALOG conserved domain and existed in OsG1/OsG1L1/2/3/4/5/6/7/8/9, and motifs 2, three and five existed in all ALOG proteins, while other motifs showed member-specific distribution. For instance, motif six just appeared in OsG1L10 and OsG1L11. Motif nine existed only in OsG1L1 and OsG1L2 and motif eight was specific to OsG1L6. These results indicated that most members of the ALOG family contained conserved motifs and members of the same clade had similar structures.

### 3.3 Collinearity analysis of ALOG gene family

**FIGURE 3:**
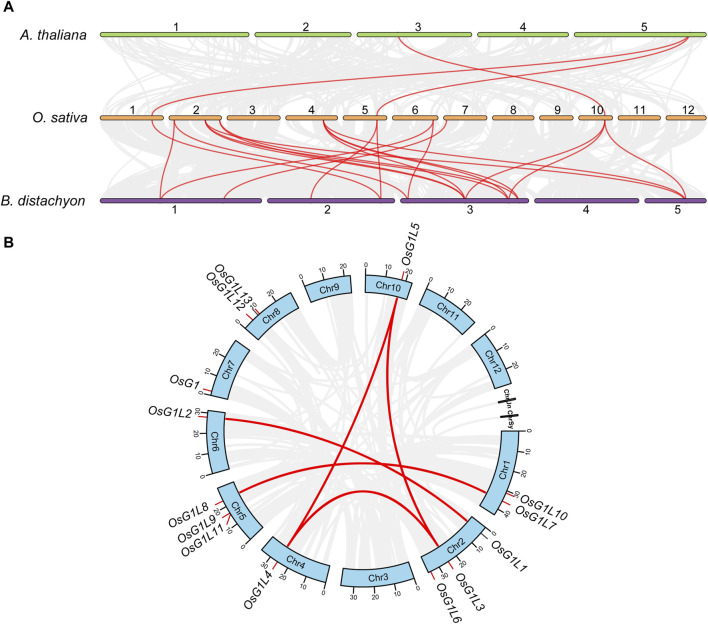
Collinearity analysis of ALOG genes. **(A)** Collinearity relationship between ALOG genes from rice with *Arabidopsis* and *Brachypodium distachyon*. The chromosomes of rice, *Arabidopsis* and *Brachypodium distachyon* were marked with different colors. **(B)** Collinearity relationship of ALOG genes from rice. The collinear relationship between the ALOG family members of different species were connected by red lines.

Gene duplication events, such as tandem and segmental duplication, provide major forces that drive the expansion of gene families and the evolution of the entire genome (Cen et al., 2023). To reveal the origin and evolutionary information of ALOG genes, a collinearity analysis was respectively performed between the genome of rice and two other species, including *Arabidopsis* (dicotyledon plant) and *B. distachyon* (monocotyledon plant) (Figure 3A). The results showed that three and 20 collinear gene pairs were found in rice with *Arabidopsis* and *B. distachyon*, respectively. Compared with the dicotyledon plant, ALOG genes revealed a high level of identity with the monocotyledon plant with fewer evolutionary separation events.

To determine the collinear relationships of the ALOG genes from rice, we used the TBtools software to examine the duplication events of ALOG members within the rice genome (Figure 3B). There were five pairs of segmental duplication genes (*OsG1L1*/*2*, *OsG1L3*/*4*/*5*, *OsG1L7*/*8*) in the ALOG gene family, and these genes were distributed on six chromosomes (chromosome 1, 2,4, 5, 6, and 10). These results suggested that segmental duplication events might be a vital driving force for the expansion of ALOG gene family.

### 3.4 Subcellular localization of OsG1L3 and OsG1L7

**FIGURE 4:**
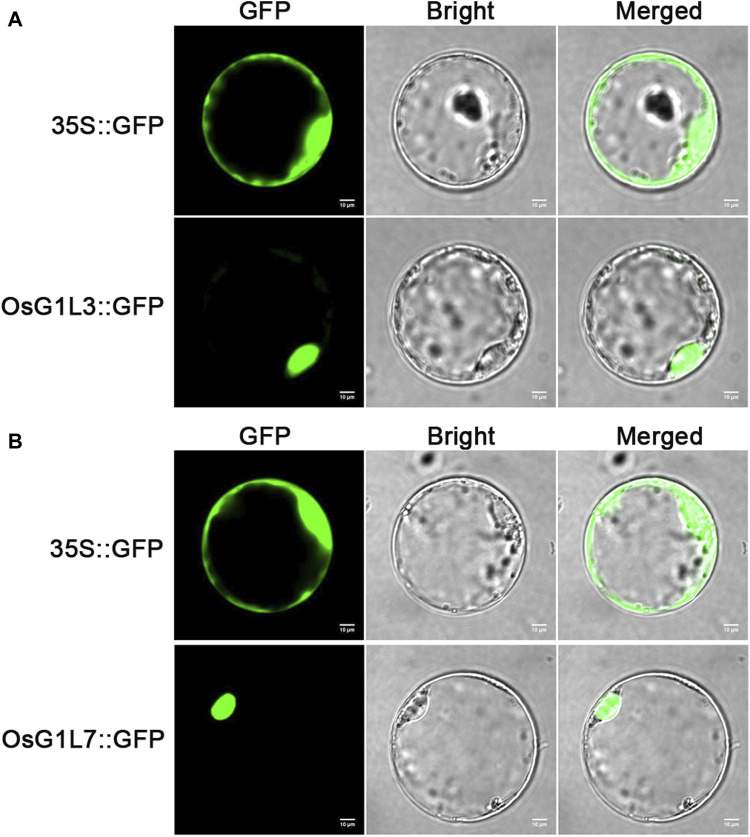
The subcellular localization of OsG1L3 and OsG1L7. **(A)** The signal of 35S::OsG1L3-GFP fusion protein; **(B)** The signal of 35S::OsG1L7-GFP fusion protein. The 35S::OsG1L3/7-GFP and control vector (35S::GFP) were transiently expressed in rice protoplasts. Confocal microscopy images were visualized after 16 h transformation. Scale bars = 10 μm.

To verify the prediction of subcellular localization, OsG1L3 and OsG1L7 were performed for subcellular localization. The full-length coding sequences of OsG1L3 and OsG1L7 were fused with green fluorescent protein (GFP) to construct the expression vectors of fusion proteins, respectively. Then, the two fusion vectors, OsG1L3-GFP and OsG1L7-GFP were transformed into rice protoplasts, and the fluorescence signals were detected by laser scanning confocal microscopy. As the results were shown in Figure 4, the fluorescence signal from 35S::GFP vector was detected throughout the whole rice protoplasts, and the signals from 35S::OsG1L3/7-GFP vector were observed in the nucleus, indicating that both OsG1L3 and OsG1L7 were localized in the nucleus (Figures 4A,B). The results of subcellular localization were consistent with the predictions (Supplementary Table S6), suggesting that ALOG genes might play crucial roles in the nucleus.

### 3.5 Expression characteristics analysis of ALOG genes in different tissues

**FIGURE 5:**
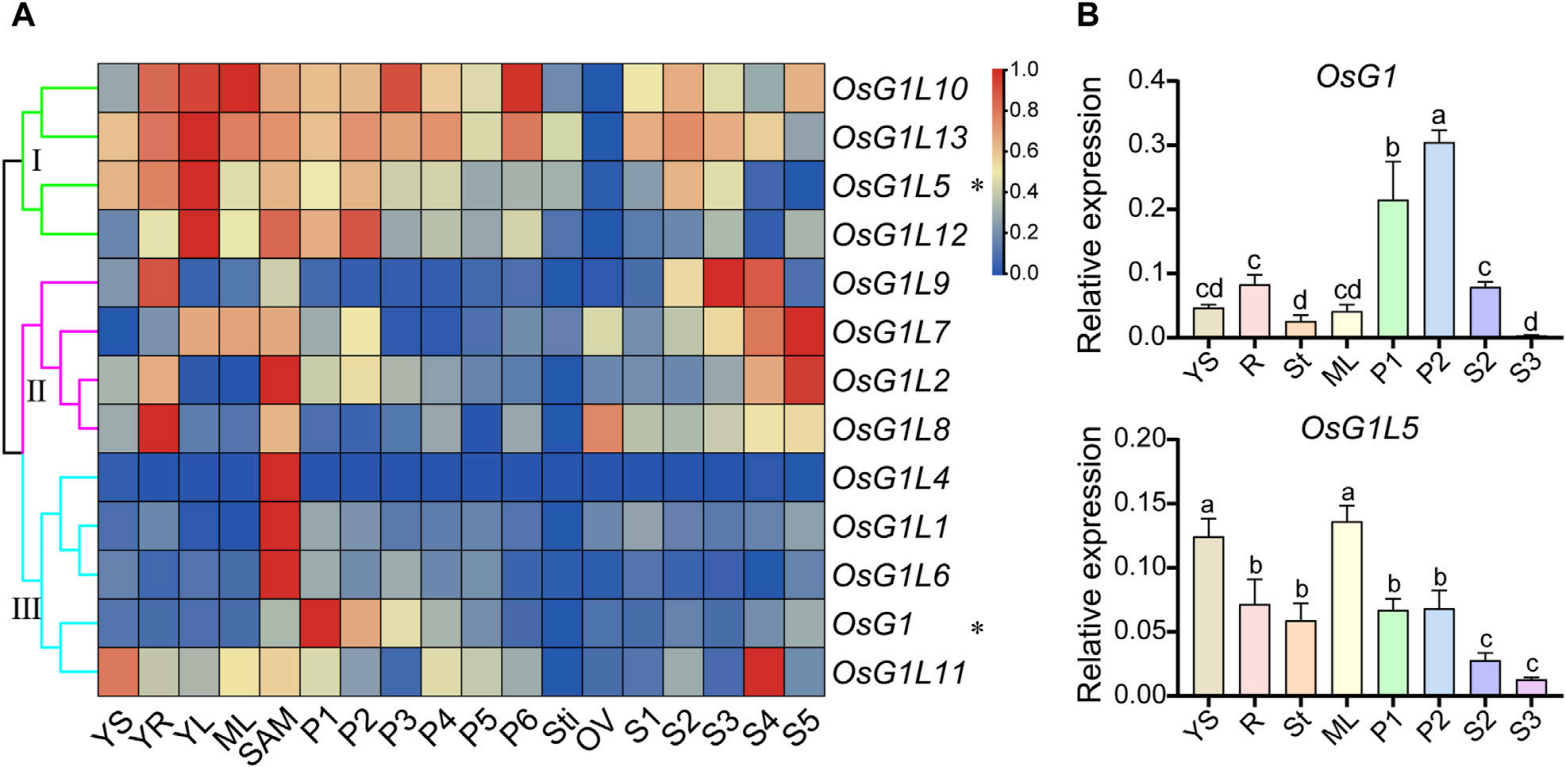
Expression profiles of ALOG genes in various tissues. **(A)** The expression of microarray data of ALOG genes in various organs at different stages. The heat map representing hierarchical cluster was generated by using the average log2 expression values of ALOG genes in various organs, including YS, 7-day-old seedlings; YR, roots from 7-day-old seedlings; YL, leaves from 7-day-old seedlings; ML, mature leaf; SAM, shoot apical meristem; different stages of panicle development: P1, 0–3 cm; P2, 3–5 cm; P3, 5–10 cm; P4, 10–15 cm; P5, 15–22 cm; P6, 22–30 cm; Sti, stigma of mature pistil; OV, mature ovary; different stages of seed development: S1, 0–2 dap (day after pollination); S2, 3–4 dap; S3, 5–10 dap; S4, 11–20 dap; S5, 21–29 dap. The differential expression of ALOG genes in various organs were consistent with the results of qRT-PCR analysis, which were represented by the asterisk (*) on the right. The color scale (representing the average log signal value) was displayed on the right side. **(B)** The expression of ALOG genes (marked by asterisks in A) in different organs using qRT-PCR, which were consistent with microarray data using qRT PCR. The error bar represented the standard deviation of three independent biological replicates (*n* = 3). Different letters (a, b, c, d) denoted significant differences at *P* < 0.05 according to ANOVA in combination with Duncan’s multiple range test.

To explore the expression patterns of ALOG genes in different tissues and organs from rice, the microarray data of 13 ALOG genes (*OsG1L3* was not detected) were collected from the Rice Functional Genomic Express Database (http://signal.salk.edu/cgi-bin/RiceGE) (Supplementary Table S7). The various tissues at different developmental stages were examined, including 7-day-old seedlings (YS), young roots (YR), young leaves (YL), mature leaves (ML), shoot apical meristem (SAM), panicles (P1-6), stigma of mature pistil (Sti), mature ovary (OV) and seeds (S1-5). The expression heatmap of ALOG genes were also constructed by TBtools (Figure 5A). The results revealed that ALOG genes mainly display three expression patterns among various tissues. For instance, group Ⅰ (*OsG1L5*/*10*/*12*/*13*) were highly expressed in seedling tissues (YS, YR, YL) and panicles (P1-6), group Ⅱ (*OsG1*/*OsG1L1*/*4*/*6*/*11*) were principally expressed in the middle and late stages of seed development (S3, S4, S5), and group Ⅲ (*OsG1L2*/*7*/*8*/*9*) were mainly expressed in shoot apical meristem (SAM) and early stages of inflorescence development (P1, P2). Most ALOG genes mainly expressed in early seedling and inflorescence suggest that these genes might paly roles in reproductive development.

To validate the expression profiles of ALOG genes in different tissues, the qRT-PCR analysis was performed in rice. The results showed that *OsG1* was highly expressed in P1 and P2, and *OsG1L5* was predominantly expressed in young seedlings and leaves, which were consistent with results from microarray data (Figure 5B). Moreover, *OsG1L1* and *OsG1L2* were highly expressed in the early inflorescence (P1), and *OsG1L6* and *OsG1L13* were specially expressed in P2, whereas, *OsG1L3* and *OsG1L4*, were preferentially expressed in ML, and *OsG1L12* were specially expressed in St (Supplementary Figure S2). These data suggested that ALOG genes might play various roles in the specific vegetative and reproductive tissues at different development stages.

### 3.6 Analysis of expression characteristics of ALOG genes in inflorescence at different stages

**FIGURE 6:**
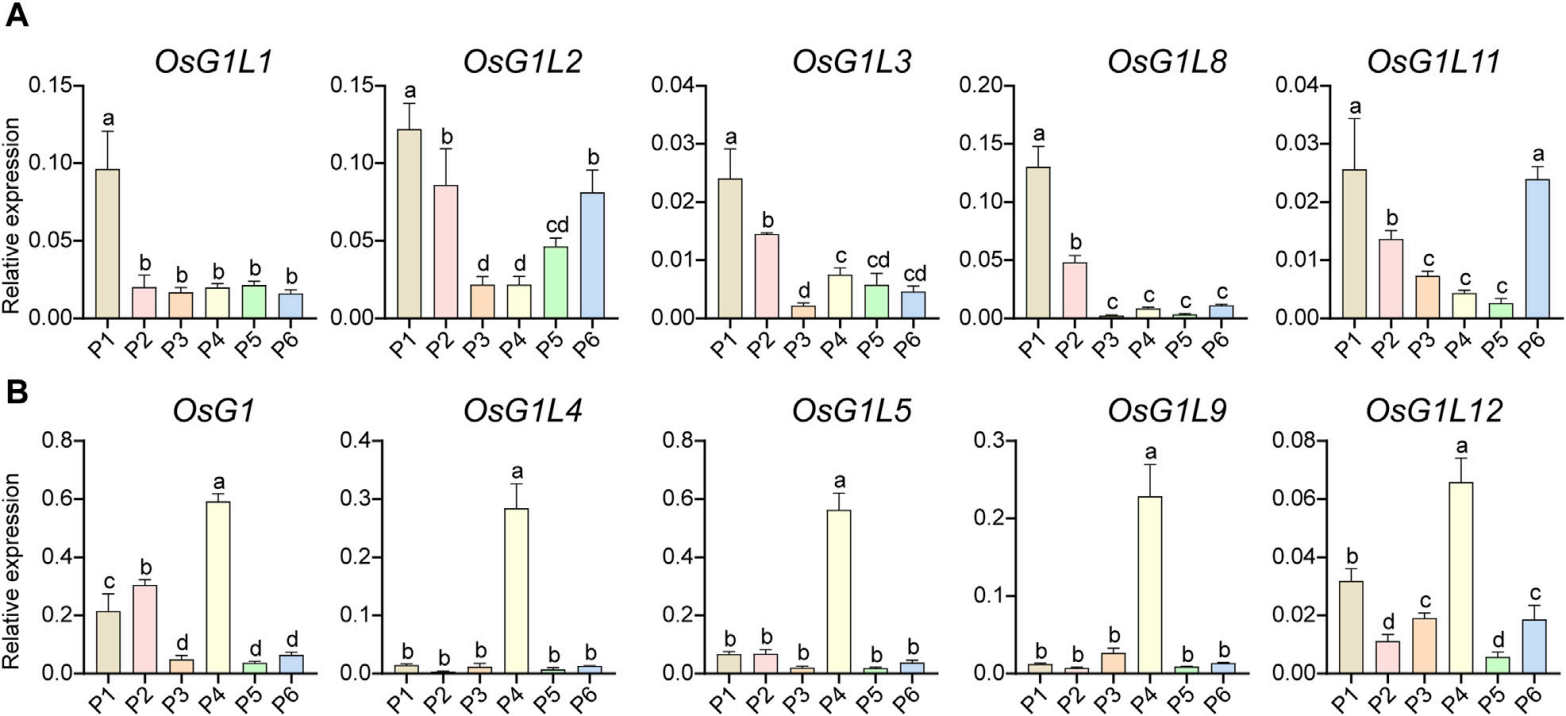
The expression profiles of ALOG genes in inflorescence. **(A)** The ALOG genes mainly expressed at P1 stage of inflorescence. **(B)** The ALOG genes predominately expressed at P4 stage of inflorescence. The stages of P1–P6 were represented by the spikelet length of 0–1, 1–3, 3–5, 5–10, 10–15, 15–22 cm. The y-axis was used to detect the relative expression level of the genes at different stages of inflorescence using qRT-PCR. The error bar represented the standard deviation of three independent biological replicates (*n* = 3). Different letters (a, b, c, d) denoted significant differences at *P* <0.05 according to ANOVA in combination with Duncan’s multiple range test.

To find the key genes of ALOG family that might have functions during the reproductive development, the expression profile analysis of rice ALOG genes in inflorescence at specific developmental stages was performed using qRT-PCR. According to the length, the inflorescences were divided into six developmental stages (P1-6). *OsG1L1*/*2*/*3*/*8*/*11* showed similar expression patterns, which were mainly expressed in the early development stages of inflorescence (P1, panicle length = 0–1 cm, Figure 6A). *OsG1*/*OsG1L4*/*5*/*9*/*12* were principally expressed at the P4 stage (panicle length = 5–10 cm, Figure 6B). In particular, *OsG1L6* was highly expressed at the P2 (panicle length = 1–3 cm) and P4 stages, *OsG1L7* was specifically expressed at the P6 (panicle length = 15–20 cm) stage, and *OsG1L13* was mainly expressed at the P2 stage (Supplementary Figure S3). These data suggested that the ALOG genes might play a variety of roles during inflorescence development in rice.

### 3.7 *Cis*-acting elements analysis in the putative promoter region of ALOG genes and expression analysis under ABA and drought treatments

**FIGURE 7:**
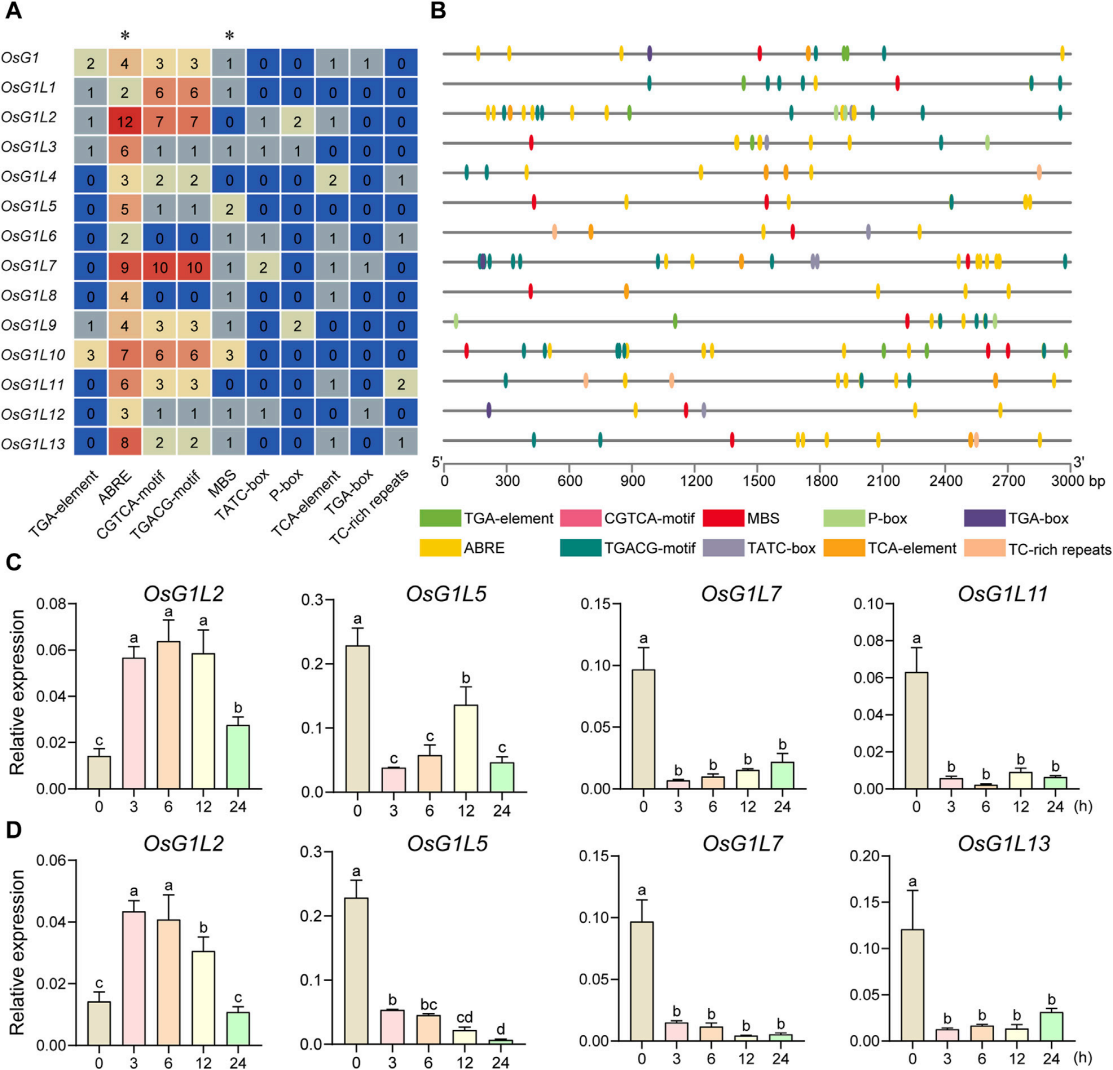
Prediction of *cis*-acting elements related to hormone and abiotic stress responses in the promoter region and expression analysis of ALOG genes in rice. **(A)** The number of cis-acting elements detected in the putative promoter region (the sequence was retrieved from the 3 kb region upstream of the start codon). The *cis*-acting elements were divided into 10 categories. **(B)** The number, type and location of stress-related and hormone-responsive elements in the putative promoter regions were speculated by the ALOG genes in **(A)**. TGA-element and TGA-box, Auxin responsive elements; ABRE, ABA responsive elements; CGTCA-motif and TGACG-motif, MeJA responsive elements; MBS, drought responsive elements; TATC-box and P-box, GA responsive elements; TCA-element, SA responsive elements; TC-rich repeats, defense and stress responsive elements. Different types of elements were represented by different colors. **(C)** The expression analysis of ALOG genes under ABA treatment. **(D)** The expression analysis of ALOG genes under drought treatment. The y axis was the relative expression level of the genes compared to Actin under different treatments using qRT-PCR. The expression level was based on the relative expression level of Actin at 0 h as a reference. The error bar represented the standard deviation of three independent biological replicates (*n* = 3). Different letters (a, b, c, d) denoted significant differences at *P* <0.05 according to ANOVA in combination with Duncan’s multiple range test.

*Cis*-acting elements within promoters of ALOG genes were predicted, so as to speculate on the possible factors affecting ALOG gene expression and the regulatory pathways in which ALOG genes might be involved. The 3,000 bp upstream genomic sequences from the start codon of 14 ALOG genes in rice were extracted by TBtools and used to predict the *cis*-acting elements in the putative promoter regions by PlantCARE (Lescot et al., 2002). A total of 216 *cis*-acting elements were detected in the putative promoter regions of the ALOG genes, including auxin responsive elements (TGA-element and TGA-box), ABA responsive elements (ABRE), MeJA responsive elements (CGTCA-motif and TGACG-motif), GA responsive elements (TATC-box and P-box), drought responsive elements (MBS), SA responsive elements (TCA-element) and defense and stress responsive elements (TC-rich repeats). The *cis*-acting elements were mainly associated with phytohormone and stress responses (Figures 7A,B). All the 14 ALOG genes contained more than two ABA responsive elements, and seven of them contained more than five ABA responsive elements. Most of the ALOG genes contained drought responsive elements. The above results imply that ALOG genes might be involved in plant response to abiotic and hormones stresses.

To explore the potential involvement of ALOG genes in abiotic stresses, the expression profile of ALOG genes under ABA and drought treatments were evaluated using qRT-PCR. The results showed that 10 genes (*OsG1L1*/*3*/*4*/*5*/*7*/*8*/*9*/*11*/*12*/*13*) were significantly decreased at 3/6/12/24 h after ABA treatment, and *OsG1L2* was increased significantly at 3/6/12 h (Figure 7C; Supplementary Figure S4). Under drought treatment, 11 genes (*OsG1L1*/*3*/*4*/*5*/*6*/*7*/*8*/*9*/*11*/*12*/*13*) were significantly decreased at 3/6/12/24 h (Figure 4D; Supplementary Figure S5). Interestingly, most of ALOG genes (*OsG1L1*/*3*/*4*/*5*/*7*/*8*/*9*/*11*/*12*/*13*) showed similar response patterns under ABA and drought stresses. The trends in expression of ALOG genes suggested that they might be involved in plant early response to ABA and drought stresses.

## 4 Discussion

### 4.1 Characterization of ALOG family members in rice

The ALOG domain had been proposed to be originated from the XerC/D-like recombinases of a new category of DIRS-1-like retroposons and consisted of a full α-helix domain with four conserved helices (Helix-1/2/3/4) and a conserved predicted zinc band (ZnR) insert (Iyer and Aravind, 2012). In our investigation, the prediction of protein 3D structure showed that Helix-1 and Helix-2 were incomplete in OsG1L11/12/13. Helix-1/2 were also missing in the amino acid sequence alignment of OsG1L10, but the 3D structure of the protein showed that its four helix structures were parallel to each other. Iyer and Aravind (2012) inferred that the helix-1 and helix-3 of the ALOG domains, which were orthogonally positioned with respect to each other, were likely to make key backbone and based contacts in the major groove. The change of helix structures might be one of the reasons for the functional diversity of ALOG members.

Based on previous studies, it has been understood that ALOG genes played an important role in plant morphogenesis and organ development (Turchetto et al., 2023). Which organs the ALOG family played roles in, however, still remained largely unclear. Currently, the systematic phylogenetic analysis of ALOG family members suggested that rice ALOG members were unevenly distributed among four clades (Li et al., 2019). In this study, the phylogenetic analysis showed that rice ALOG members could be divided into six clades (A, C, D, E, H and I): the numbers in clade D, and H were consistent with the results of Li et al. (Li et al., 2019), including OsG1 and OsG1L7/8/9, respectively; clade A included OsG1L2/10/11/12/13, clade C included only OsG1L1, clade E included OsG1L3/4/5 and clade I included OsG1L6, which were different from the previous findings. The different distribution of rice ALOG members in the phylogenetic trees might be caused by the different plant species. Among the members of the ALOG family in rice, *OsG1L1*/*2*/*5*/*6* had been reported to be involved in the regulation of rice reproductive development. The mutant plants of *OsG1L1*/*2, osg1l1*/*osg1l2,* developed panicles that were shorter, with fewer primary branch meristems (PBMs), extremely reduced secondary branch meristems (SBMs) and fewer spikelets than the wild-type plants (Beretta et al., 2023). In the dominant gain-of-function mutant *tawawa1-D* (the mutant plant of *OsG1L5*), the activity of the inflorescence meristem (IM) was extended and spikelet specification was delayed, resulting in prolonged branch formation and increased numbers of spikelets (Yoshida et al., 2013). Abnormal floral organs were observed in the *afd1* (the mutant plant of *OsG1L6*), including slender and thick hulls, and hull-like lodicules (Ren et al., 2016). It was especially noteworthy that OsG1L1/2/5/6 were distributed in four different clades in the phylogenetic tree, suggesting that they might be conservative in function.

In order to further explore the conservatism of the members of the ALOG family, collinearity analysis was performed on 14 rice ALOG members. Intraspecific collinearity analysis showed that half of the members of the rice ALOG family (*OsG1L1*/*2*, *OsG1L3*/*4*/*5*, *OsG1L7*/*8*) had collinearity. Although *OsG1L1*/*2* did not belong to the same clade, they had a collinear relationship, further confirming that rice ALOG family members might be functionally conserved. *OsG1L3*/*4*/*5* had collinear relationships and were also in the same clade in the phylogenetic tree, hinting that *OsG1L3*/*4* might have similar functions to *OsG1L5* in rice.

### 4.2 Differential expression patterns of ALOG genes in different tissues

ALOG family members had been reported in a few plants and played important roles in plant growth and development, particularly in reproductive development (Chen et al., 2019). The analysis on spatial and temporal expression patterns of genes might provide useful information for establishing their putative functions (Zhiguo et al., 2015). In our study, the analysis of microarray data showed that 10 of the 13 genes were specifically expressed during the shoot apical meristem (SAM) period (Figure 5A). Previous studies had shown that *OsG1L1*/*2*/*5* were preferentially expressed in the IM tissues, indicating that they play an important role in reproductive meristems, especially in inflorescences (Beretta et al., 2023). The qRT-PCR data showed that *OsG1L1*/*2*/*5* were specifically expressed in the early stage of inflorescence development (P1, P2) (Figure 5B). These results suggested that they might regulate inflorescence development from the SAM period to the early IM period. Similarly, the microarray data showed that *OsG1L6* was preferentially expressed at the SAM stage, and qRT-PCR data showed that it was preferentially expressed at the P2 stage. According to recent studies, *OsG1L6* was expressed in the 4–5 cm period of panicle length, and its pleiotropic effect affects the development of spikelets (Peng et al., 2017). These data indicated that *OsG1L6* might regulated spikelet development similar to *OsG1L1*/*2*/*5*. Most ALOG genes were specifically expressed in the SAM stage, and half of the genes were highly expressed in the early stage of inflorescence development, suggesting that these genes also played a role in the inflorescence development.

Inflorescence development is a relatively long and complex process. We performed expression analysis for a more comprehensive inflorescence development period (P1∼6). The results showed that *OsG1L1*/*2* were highly expressed at the P1 stage, *OsG1L5* was specifically expressed at the P4 stage, and *OsG1L6* was specifically expressed at the P2 and P4 stages (Figures 6A,B). According to previous studies, *OsG1L6* not only regulates inflorescence development, but also affects grain size (Peng et al., 2017). The research proposed that *OsG1L5* regulates the inflorescence architecture of rice through the promotion of IM activity and suppression of the phase change to SM identity. The similar expression pattern of *OsG1L5* and *OsG1L6* produced functional differentiation, indicating that they might regulate inflorescence development in different ways.

### 4.3 Potential involvement of rice ALOG genes in the regulation of abiotic stress and hormone responses

Drought is a major environmental factor that affects the development of plants, in addition, abscisic acid (ABA), which is the central regulator of abiotic stress resistance in plants, coordinates an array of functions enabling plants to cope with different stresses. Abiotic stresses, especially drought, induce ABA accumulation, which triggers rapid biochemical and physiological responses that enhance stress adaptation. At present, there were still limited reports on abiotic stress of ALOG family members in rice (Xu et al., 2020).

The regulation of gene expression by *cis*-elements in the promoter region had become the important adaptive mechanism for organisms to respond to environmental changes (Walther et al., 2007; Lai et al., 2022). We conducted in-depth analysis of the upstream promoter region of the ALOG genes in rice. The ABA response elements (ABRE) were abundant in the promoter region of the ALOG genes, and half of the ALOG members contained at least 5 ABRE elements, of which the most contained 12 ABRE elements. Drought responsive elements (MBS) closely related to ABA also existed in the promoter regions of most ALOG members. (Figure 7A). The expression analysis using real-time PCR data revealed that most ALOG genes showed downregulated expression levels under ABA and drought stresses (Figures 7C,D). A recent research had shown that the expression level of *LSH8* decreased under ABA treatment. *LSH8* mutant lines *lsh8-1* and *lsh8-2* showed ABA-insensitive phenotypes during seed germination, primary root and lateral root development. The ABA response elements (ABRE) were also identified in the *LSH8* promoter region and the protein localized in the nucleus (Zou et al., 2021). In this study, *OsG1L7* also contained a large number of ABRE elements (9), and subcellular localization results also showed that it was located in the nucleus (Figure 4B). Similarly, *OsG1L7* was significantly downregulate under ABA and drought treatment, suggesting that *OsG1L7* might have similar functions to *LSH8* and might also be an important factor in the ABA signaling pathway. Interestingly, *OsGlL2* and *OsGlL7* had similar *cis*-element combination. *OsG1L2* had the most 12 ABRE elements, and *OsG1L7* had 9 ABRE elements next to OsG1L2. However, *OsGlL2* and *OsGlL7* showed opposite expression patterns under ABA and drought stresses. These results suggested that they might respond to aboitic stresses in different ways. It was also worth noting that the expression levels of most ALOG genes were significantly decreased under ABA and drought treatments, indicating that rice ALOG family genes might be involved in the regulation of abiotic stress. However, the molecular mechanism of ALOG family genes involved in abiotic stress regulation is still unclear.

## 5 Conclusion

ALOG is an important family that plays a role in plant growth and development. In this study, based on the analysis of phylogenetic relationship, protein 3D structure, collinearity, *cis*-acting elements, and expression profile, it is concluded that the ALOG family in rice have conserved domain, the *cis*-acting elements in the promoters are mainly involved in the response to hormone and abiotic stresses, and the most genes are predominantly expressed in vegetative tissues and early inflorescences. These data could provide important insights for further understanding the functions of ALOG family genes in rice growth and development, and offer valuable information for the selecting candidate genes and functional validation studies of rice ALOG members.

## Data Availability

The datasets presented in this study can be found in online repositories. The names of the repository/repositories and accession number(s) can be found in the article/Supplementary Material.
