# Corrigendum: Expression profiling of ALOG family genes during inflorescence development and abiotic stress responses in rice (*Oryza sativa L.*)

**DOI:** 10.3389/fgene.2024.1419283

**Published:** 2024-05-03

**Authors:** Zhiyuan Liu, Zhenjiang Fan, Lei Wang, Siyue Zhang, Weichen Xu, Sijie Zhao, Sijia Fang, Mei Liu, Sackitey Mark Kofi, Shuangxi Zhang, Ningning Kang, Hao Ai, Ruining Li, Tingting Feng, Shuya Wei, Heming Zhao

**Affiliations:** ^1^ Center for Crop Biotechnology, College of Agriculture, Anhui Science and Technology University, Fengyang, China; ^2^ College of Bioengineering, Wuhan Polytechnic University, Wuhan, China

**Keywords:** rice, ALOG family, expression analysis, inflorescence development, abiotic stress

In the published article, there were errors in affiliations 1 and 2. Instead of “^1^Country Center for Crop Biotechnology, College of Agriculture, Anhui Science and Technology University, Fengyang, China”, it should be “^1^Center for Crop Biotechnology, College of Agriculture, Anhui Science and Technology University, Fengyang, China”. Instead of “^2^College of Bioengineering, Wuhan Polytechnic, Wuhan, China”, it should be “^2^College of Bioengineering, Wuhan Polytechnic University, Wuhan, China”.

The authors apologize for these errors and state that this does not change the scientific conclusions of the article in any way. The original article has been updated.

