# Supplementary material for: Expression profiling of ALOG family genes during inflorescence development and abiotic stress responses in rice (Oryza sativa L.)
Source: Front Genet. 2024 Apr 8;15:1381690. doi: 10.3389/fgene.2024.1381690 (PMC11033443; doi:10.3389/fgene.2024.1381690)
Supplement: Supplementary file 1 [file DataSheet1.ZIP › ALOG in rice -Figure Legend and Supplementary Files/01-Table S1.docx]

Table S1. Full-length protein sequence of 59 ALOG family members in rice, *Arabidopsis thaliana*, *Brachypodium distachyon*, *Solanum lycopersicum*, and *Sorghum bicolor*.

>OsG1

MSSSSAAALGSDDGCSPAELRPSRYESQKRRDWQTFTQYLAAHRPPLELRRCSGAHVLEFLRYLDRFGKTRVHEPPCPSYGGRSPSAAGPVAAAAAACQCPLRQAWGSLDALVGRLRAAYDERHGRAGEPDAVAGAGAVATDSTSSSSAAAANPFAARAVRLYLRDVRDAQAMARGISYHKKKKRRGGNMNGARGGGGGGARAGVNDGDATAPPVAVTPGLPLPPLPPCLNGVPFEYCDFGSVLGGAHGAHGGHGGGGGGFYGAGVYLPFLYNTFS

>OsG1L1

MDMIGMASPAESPGGGGTARPSRYESQKRRDWQTFGQYLRNHRPPLELSRCSGAHVLEFLRYLDQFGKTKVHAHGCPFFGHPSPPAPCPCPLRQAWGSLDALVGRLRAAFEEHGGRPESNPFGARAVRLYLRDIRDTQSKARGIAYEKKRRKRAAASHTKQKQQQQQLVEQAVAPPAAAAAAAALPDMETTTTTTTVPHFLFPAHFLHGHYFLAPAGEQPGGGDVAASTGGAAGAPSGGGGEDLVLAMAAAAAAAEAHAAGCMMPLSVFN

>OsG1L2

MQGGGGGDSSGGGGGEAPRPSRYESQKRRDWHTFGQYLRNHRPPLELSRCSGAHVLEFLRYLDQFGKTKVHAAGCPFFGHPSPPAPCPCPLRQAWGSLDALVGRLRAAFEEHGGRPEANPFGARAVRLYLREVRDSQAKARGIAYEKKRRKRPPTSSSSSQAAAAAAAATSPASPAASPTPPPPPPTERSADVRPMPPEGHFFIPHPHFMHGHFLVPGGDADHHHQVSNAGNGGNTNTNTNTNTGGGGGNGDEMAVAMAAVAEAHAAGCMLPLSVFN

>OsG1L3

MELSPPNHESSPPTAGGGGGGGGDGAGGSSSAGASSSAGGGAATPQTPSRYEAQKRRDWNTFGQYLRNHRPPLGLAQCSGAHVLEFLRYLDQFGKTKVHTAACPFFGHPNPPAPCPCPLRQAWGSLDALVGRLRAAFEENGGRPESNPFAVRAVRLYLREVREHQARARGVSYEKKKRKKPQPADTSGGGGHPHPPPPPPPPPSAGAAC

>OsG1L4

MDLSPNPDSPPSGGGNGGGGGSSSSNSSPSMGAGAPQSPSRYEAQKRRDWNTFGQYLRNHRPPLSLAQCSGAHVLEFLRYLDQFGKTKVHTAACPFFGHPSPPAPCPCPLRQAWGSLDALVGRLRAAFEENGGRPESNPFAARAVRLYLREVREHQARARGVSYEKKKRKKPQQQQLQGGDSSGLHGHQHHPPPPPPAGAAC

>OsG1L5

MEFVAHAAAPDSPHSDSGGGGGGMATGATSASAAGASPSRYESQKRRDWNTFGQYLRNHRPPLSLARCSGAHVLEFLRYLDQFGKTKVHAPACPFFGHPAPPAPCPCPLRQAWGSLDALVGRLRAAYEENGGRPENNPFGARAVRLYLREVREHQARARGVSYEKKKRKKPPHPSSAAAAHDDAANGALHHHHHMPPPPPGAAA

>OsG1L6

MDRHHHHHHHHHHHMMSGGGQDPAAGDGGAGGATQDSFFLGPAAAAMFSGAGSSSSGAGTSAGGGGGGPSPSSSSPSLSRYESQKRRDWNTFGQYLRNHRPPLSLSRCSGAHVLEFLKYMDQFGKTKVHTPVCPFYGHPNPPAPCPCPLRQAWGSLDALIGRLRAAYEENGGTPEMNPFGARAVRLYLREVRETQARARGISYEKKKRKKPSSAGAGAGPSSEGSPPPPGGSASGGGDTSASPQFIIP

>OsG1L7

MDPSGPGPSSAAAGGAPAVAAAPQPPAQLSRYESQKRRDWNTFLQYLRNHRPPLTLARCSGAHVIEFLRYLDQFGKTKVHASGCAFYGQPSPPGPCPCPLRQAWGSLDALIGRLRAAYEESGGTPESNPFAARAVRIYLREVRDSQAKARGIPYEKKKRKRSQAAQPAGVEPSGSSSAAAAAAGGGDAGSGGGAAATTTAQPGGSGTAPSAS

>OsG1L8

MEGGGGGADGQAQPVAQAPPAMQPMQQLSRYESQKRRDWNTFLQYLKNHRPPLTLARCSGAHVIEFLKYLDQFGKTKVHASGCAYYGQPSPPAPCPCPLRQAWGSLDALIGRLRAAYEESGHAPESNPFAARAVRIYLREVRDAQAKARGIPYEKKKRKRTQQQQPPPPPPPPPQHQPGAAAGEASSSSSAAAAAVAAEGSGSSAAAAAATSQTGGGGGGSTTTTTASAAAPTTATRV

>OsG1L9

MEPSPDAPRAGAAEEQPGPSSSASAPAPAASSNEEEGRHQSQAQQQVQEAQPQPLAQQAPAAAGLSRYESQKRRDWNTFLQYLRNHKPPLTLPRCSGAHVIEFLKYLDQFGKTKVHADGCAYFGEPNPPAPCACPLRQAWGSLDALIGRLRAAYEESGGRPESNPFAARAVRIYLREVREAQAKARGIPYEKKRKRGAAAAAAAPPVVVAPPPVVTAPDDATGTSGGAGEDDDDDEATHSGEQQDTTPAASPTTPPATSVGTTTAAATAAAAKGSAAKGSATSS

>OsG1L10

MAKHTRKSFISFEPDYARFMHHHMKNASCTSFHSLTYTTRMGDTPGYEQKVYVVCFYHSVNYRVFQGNTLQQLLLRSVHLEHWGTPGYWSITLANMARTAAGRVERGGGRGGRACGRRSHPSSPAPWPCPLRQAWGSLDVLVGRLRTAFDEHGGHPEANPFGARVVRLYLREVCDSQAKVRGIAYEKKRRKRPPTSSSHSQDGTAATCPASPAASPTPLPPPPERSADMGACVAIVVAVGCTPLSLAARRGCSYCALARRR

>OsG1L11

MPLGPHISSISCLSPHLSFLSQMARTAAGRVERGGGRGGRACGHRSHPSPPTLCPCPLRQAWGSLDTLVGRLCTAFDEHGGHPEANPFGARVVRLYVRDSQAKVRGIAYEKKRRKRPPTSFSHSQAAAAATCPASPAASPTPERSADMGACVAIAVAVGCTPLSLAARRGCSYCALAYRR

>OsG1L12

MSVLSPISLLISLFSLSFLSQMARTAAGGVERGSGSGGRVRSRRSHPSPLAPCPCPLRQAWGSLDVLVGHLRAAFEEHGGHPEANPFSARAVRLYLHEVRDS

>OsG1L13

MARTVAGGVERGGGGGGRARGRRSHPSLPVPCPCLLRQAWGSLNALVGRFRAAFEEHGGQPEANPFGARAVRLYLHEVYDCQAKARGIAYEKKRWKRPPTSSSHSQAAAAATSSASQPLARCRHRRCRRDQPTWLTVPDSLMVVLCAQVQIKSKAERKRLWMLPSDSPSTGTHLTLKDSSEISENTISW

>AtLSH1

MDLISHQPNKNPNSSTQLTPPSSSRYENQKRRDWNTFCQYLRNHRPPLSLPSCSGAHVLEFLRYLDQFGKTKVHHQNCAFFGLPNPPAPCPCPLRQAWGSLDALIGRLRAAYEENGGPPEANPFGSRAVRLFLREVRDFQAKARGVSYEKKRKRVNRQKPQTQPPLQLQQQQQQPQQGQSMMANYSGATV

>AtLSH2

MDLISQNHNNRNPNTSLSTQTPSSFSSPPSSSRYENQKRRDWNTFCQYLRNHHPPLSLASCSGAHVLDFLRYLDQFGKTKVHHQNCAFFGLPNPPAPCPCPLRQAWGSLDALIGRLRAAYEENGGAPETSPFGSRSVRIFLREVRDFQAKSRGVSYEKKRKRVNNKQITQSQPQSQPPLPQQPQQEQGQSMMANYHHGATQ

>AtLSH3

MDMIPQLMEGSSAYGGVTNLNIISNNSSSVTGATGGEATQPLSSSSSPSANSSRYENQKRRDWNTFGQYLRNHRPPLSLSRCSGAHVLEFLRYLDQFGKTKVHTNICHFYGHPNPPAPCPCPLRQAWGSLDALIGRLRAAFEENGGKPETNPFGARAVRLYLREVRDMQSKARGVSYEKKKRKRPLPSSSTSSSSAVASHQQFQMLPGTSSTTQLKFEK

>AtLSH4

MDHIIGFMGTTNMSHNTNLMIAAAATTTTTSSSSSSSSGGSGTNQLSRYENQKRRDWNTFGQYLRNHRPPLSLSRCSGAHVLEFLRYLDQFGKTKVHTHLCPFFGHPNPPAPCACPLRQAWGSLDALIGRLRAAFEENGGSPETNPFGARAVRLYLREVRDSQAKARGISYEKKKRKRPPPPLPPAQPAISSSPN

>AtLSH5

MEGETAAKAAASSSSSPSRYESQKRRDWNTFLQYLRNHKPPLNLSRCSGAHVLEFLKYLDQFGKTKVHATACPFFGQPNPPSQCTCPLKQAWGSLDALIGRLRAAFEEIGGGLPESNPFAAKAVRIYLKEVRQTQAKARGIPYDKKKRKRPHTDTATPIAGDGDDAEGSGGAALVVTAATTV

>AtLSH6

MESADSGRSDPVKGDDPGPSFVSSPPATPSRYESQKRRDWNTFLQYLKNHKPPLALSRCSGAHVIEFLKYLDQFGKTKVHVAACPYFGHQQPPSPCSCPLKQAWGSLDALIGRLRAAYEENGGRPDSNPFAARAVRIYLREVRESQAKARGIPYEKKKRKRPPTVTTVRVDVASSRQSDGDPCNVGAPSVAEAVPP

>AtLSH7

MASHSNKGKGIAEGSSQPQSQPQPQPHQPQSPPNPPALSRYESQKRRDWNTFCQYLRNQQPPVHISQCGSNHILDFLQYLDQFGKTKVHIHGCVFFGQVEPAGQCNCPLKQAWGSLDALIGRLRAAFEENGGLPERNPFAGGGIRVFLREVRDSQAKARGVPYKKRKKRKKRNPMKSHDGEDGTTGTSSSSNLAS

>AtLSH8

MTSTNTRNKGKCIVEGPPPTLSRYESQKSRDWNTFCQYLMTKMPPVHVWECESNHILDFLQSRDQFGKTKVHIQGCVFFGQKEPPGECNCPLKQAWGSLDALIGRLRAAYEENGGLTEKNPFARGGIRIFLREVRGSQAKARGVLYKKKKRLVVVGTGTSTTWT

>AtLSH9

MSSDRHTPTKDPPDHPSSSSNHHKQPLPPQPQQPLSRYESQKRRDWNTFVQYLKSQNPPLMMSQFDYTHVLSFLRYLDQFGKTKVHHQACVFFGQPDPPGPCTCPLKQAWGSLDALIGRLRAAYEEHGGGSPDTNPFANGSIRVHLREVRESQAKARGIPYRKKKRRKTKNEVVVVKKDVANSSTPNQSFT

>AtLSH10

MDMIPQLMEGSSAYGGVTNLNIISNNSSSVTGATGGEATQPLSSSSSPSANSSRYENQKRRDWNTFGQYLRNHRPPLSLSRCSGAHVLEFLRYLDQFGKTKVHTNICHFYGHPNPPAPCPCPLRQAWGSLDALIGRLRAAFEENGGKPETNPFGARAVRLYLREVRDMQSKARGVSYEKKKRKRPLPSSSTSSSSAVASHQQFQMLPGTSSTTQLKFEK

>SbG1

MDLIPHPDSPHSDNSGGVGGGGGGGGVGSVSGALSPAGASSAGAVSALASPSRYESQKRRDWNTFGQYLRNHRPPLSLARCSGAHVLEFLRYLDQFGKTKVHTPACPFFGHPAPPAPCPCPLRQAWGSLDALVGRLRAAYEENGGRPENNPFGARAVRLYLREVRDHQSRARGVSYEKKKRKKAPAHPVPAAVISSSSSHDGNGHHHYEHHQMPPPPPPGAAA

>SbG2

MSTSGGAGARSWSPPRRASRYESQKRRDWHTFTRYLAAHRPPLELCRCSGAHVLEFLRYLDRFGKTRVHAPLCAAYGGGGGGPALVAAAPCQCPLRQAWGSLDALVGRLRAAFEERHGARGSGTIWTSSQSQSQSQQPAVVDGDAANPFAARAVRLYLRDVRDAQSRARGISYSRKKKKRSKQQDGAAAAAAGCARPHVNGATSLMHAAAPPPAPPPPPPPPPPPPYCLAGVPFEFCDYGTVLGGVTANGAPGFYLPSLFNTFGKLLIIYLLRSKTYYLFISLR

>SbG3

MDPSGPGPSSVMGAAGGGEAPAVAPPRPAQLSRYESQKRRDWNTFLQYLRNHRPPLTLARCSGAHVIEFLRYLDQFGKTKVHAAGCAYYGQPAPPGPCPCPLRQAWGSLDALIGRLRAAYEESGGTPESNPFAARAVRIYLREVRDSQAKARGIPYEKKKRKRAQQQQAAAAADPASTSSSAAAAGGSGTSGRAAAAAAASAAQAGGSSAAPSTT

>SbG4

MLVMAAKSSVFFLTPRRCRRTNRGGLHADLRREEQADDMQVGGGAADSPGAAAGAEAPRPSRYESQKRRDWHTFGQYLRNHRPPLELARCSGAHVLEFLRYLDQFGKTKVHAPGCPFFGHPSPPAPCPCPLRQAWGSLDALVGRLRAAFEEHGGRPEANPFGARAVRLYLREVRDSQAKARGIAYEKKRRKRPSASSSQSSPQAATTPPQQAPPVSSPALSDVVAERADVRAHVPDAGHQQHHHLHQHQHQHHFFMPHPQFLHGFSLLPGNPEAVAANGNGGGSSSASVAAGNGDEIALAMAAAAEAHAAGCMLPLSVFN

>SbG5

MEFAGGGIAAPAADSPGAGASRPSRYESQKRRDWHTFGQYLRNHRPPLELPRCSGAHVLEFLRYLDQFGKTKVHASGCPFFGHPSPPAPCPCPLKQAWGSLDALVGRLRAAFEEHGGRPEANPFGARAVRLYLREVRDSQAKARGIAYEKKRRKRHPAAHRQPKQQQDGHGQHHHPSQAAPGPVAERRLADVAEPPAPHFLIPHAHFLHGHFLAPVTQPIDPAAGGGGGGAGEDIVLAMAAAAEAHAAGFFMPLSVFH

>SbG6

MDLSPNPESPGGGGDGGGGGGGAGGSSSGPSSSSAQGGGTPQTPSRYEAQKRRDWNTFGQYLRNHRPPLSLAQCSGAHVLEFLRYLDQFGKTKVHTAACPFFGHPNPPAPCPCPLRQAWGSLDALVGRLRAAFEENGGRPESNPFAARAVRLYLREVREHQARARGVSYEKKKRKKAQPPDHASGSGGQGPHHHHPPPPAPPSAGAAC

>SbG7

MDHHHHHHHHHHHHMIPGQEPSAADGAAPDSFFLGPAAAVIFPGGAGASGAGSSSSGAAALGSSVGGGGGPSPSSSSPSLSRYESQKRRDWNTFGQYLRNHRPPLSLSRCSGAHVLEFLKYMDQFGKTKVHTPVCPFYGHPNPPAPCPCPLRQAWGSLDALIGRLRAAYEENGGTPEMNPFGARAVRLYLREVRETQARARGISYEKKKRKKPSAASAAAAGPSSEGSPPPGPSGGGGGPDTSVSPQFIMP

>SbG8

MKHIILMYWVYMLHNLCRWHKKSELDEVAAVVEEVSPSAQHQEAPAPPVSRFPQAQPQPLAQQPLVPSPLGLSRYESQKWRDWNTFLQYLQNHRPPLKLACCTGAHVIEFLRYLDQFGKTRVHLEGCDYFGQPNPPVPCACPLRQAWGSLDALIGRLRAGYEEFGGRPESNPFMAKDVRIYLRDVREAQAKASGISYVKKKPKHGSTAASPVAPPPVVTAETAGTMSGAAGEVDDDDEPSSSVGELRQRQRKVATSAGGIPAPAAAARAAQQPPTHAELERVRCVGSGAGGTVWMVRHRGTGQLYALKVLKGNHNYDVRRQIAREIAILRTADHPAVVRCHGMYEHGGELQILLEYMDGGSLNGHHIATEPLLADVARQVLSGIAYLHRRHIVHCGIKPSNLLIDSARHVKIAEFGVGHILKQTMEPSNSSVGTIAYMSPEQINTNLSDGSYAGDVWSFGLSILELYLGRFPFGENENLSKQGDLANLMCAICFSYPPEPPRTASPEFRGFISCCLKKNPAKRLTAAQLLQHPFVASTQPQPQLLAAPPLP

>SbG9

MDMSPNPDSPSSGGGNGIGPSSGGASPSVGSMTAPQSPSRYEAQKRRDWNTFGQYLRNHRPPLSLAQCSGAHVLEFLRYLDQFGKTKVHGPACPFFGHPNPPAPCPCPLRQAWGSLDALVGRLRAAFEENGGRPESNPFAARAVRLYLREVREHQARARGVSYEKKKRKKPQQLPGDSSGGLHGHTHQPPPPPPAGAAC

>SbG10

MEPGPDAPAGGGGTSSSAPAETGPSSSSAAAAAAASSSSNRQQAAEQEAAPQQQAGAQQPQRQQPAAAPPAQPQAQQPQPLAQQPPPPPPPPAGLSRYESQKRRDWNTFLQYLRNHKPPLTLARCSGAHVIEFLRYLDQFGKTKVHAEGCAYFGQPNPPAPCACPLRQAWGSLDALIGRLRAAYEESGGRPESNPFAAKAVRIYLRDVREAQAKARGIPYEKKKRKRGSAAAPPVAPPPVVTAGTTSGAAGGEEEEDDDDEPSPSAAGERPQQQTTTPASASASAPPPAASTSSASASSSTAATATATVTTTTTRKEEEGSAPSS

>SbG11

MDPSGPAAGPSSSAARGGGDDDAHAPPPPQQQHHQVQPLAQAQPQQAAAAQPHQAPPPPQQQLSRYESQKRRDWNTFLQYLRNHRPPLTLARCSGAHVIEFLKYLDQFGKTKVHAAGCAYFGQPNPPAPCPCPLRQAWGSLDALIGRLRAAYEESGHAPESNPFAARAVRIYLRDVRDAQAKARGIPYEKKSRKRKQPAAGSGEASSSSAAAAAREAGAAGDGSGGSAAATKAAPTTGQGSGTTAAAAAAPTSTSRV

>SbG12

MQVGGGAADSPGAAAGAEAPRPSRYESQKRRDWHTFGQYLRNHRPPLELARCSGAHVLEFLRYLDQFGKTKVHAPGCPFFGHPSPPAPCPCPLRQAWGSLDALVGRLRAAFEEHGGRPEANPFGARAVRLYLREVRDSQAKARGIAYEKKRRKRPSASSSQSSPQAATTPPQQAPPVSSPALSDVVAERADVRAHVPDAGHQQHHHLHQHQHQHHFFMPHPQFLHGFSLLPGNPEAVAANGNGGGSSSASVAAGNGDEIALAMAAAAEAHAAGCMLPLSVFN

>SlG1

MLDVYSTINSVSQNFSLSSAPAPTLPLPPPSSPPTVSRYELQKRRDWNTFGQYLRNHKPPLILARCSGANILEFLKYLDQFGKTKVHSCNCPFFGDPHPPAPCNCPLKQAWGSLDALIGRLRAAFEENGGRTETNPFGARAVRLYLKEVRDTQAKARGIAYEKKKRRNIKQRISSTINNCD

>SlG2

WTERCDFHLFFFLQTAPIPFSFPSSVSLFFSFKKKMNPSTIVMTKELSAGSSRSGGEQLQNNNPAPLSRYESQKRRDWNTFGQYLKNQRPPVPLSQCNCNHVLEFLRYLDQFGKTKVHLHGCVFFGQPDPPAPCTCPLRQAWGSLDALIGRLRAAYEENGGSPENNPFGNGAIRLYLREVKECQAKARGIPYKKKKKRKLNNNSIKPIGAGVGASADQHKNLMQANI

>SlG3

MDVANDNPSNDVVDILSTPRLSRYESQKRRDWNTFCQYIRNHHPLMSLLQCSSIHVLEFLRYLDQFGKTKVHNSNCPFFGMINPPAPCACPLRQAWGSLDALIGRLRAAYEEHGGNSEMNPFGARSIKLFLRDVRNFQSKSRGISYDKKRKRSKRNHKNIMEMKEVDHQIHDDKNVGANL

>SlG4

MDFVTAQGNNFTTSGNNMVQGTNFIANSTTMIESSVPPLSRYENQKRRDWNTFCQYVRNHQPPLSLPQCTSAHILEFLRYLDQFGKTKVHNQNCPFFGLLNPPAPCPCPLRQAWGSLDALIGRLRAAYEENGGKPEMNPFGSRNVRLFLREVRDFQSKSRGVSYEKKRKRTTSSTNNNKSKIITVIDGGGDGCGTGTCATFCGYGNIGNGN

>SlG5

MDSTSRVEQPDPNIVGSSEGGTGTSSASAVTEGGQSTTVSAAPPSRYESQKRRDWNTFLQYLRNHKPPLTLARCSGAHVIEFLKYLDQFGKTKVHVTGCPYFGHPNPPAPCACPLKQAWGSLDALIGRLRAAYEENGGKPESNPFGAKAVRIYLREVRESQAKARGIPYEKKKRKRPSTSSSVATAGSAVAAEGGSSSGGGDGSGGDGVIGQQPPTDPNTTV

>SlG6

MASFTELVESSNHHHHEKINIETVNNIEIISVSASSSSSAATPAPASSSSRYENQKRRDWNTFGQYLRNHRPPLTLSRCSGAHVLEFLRYLDQFGKTKVHTPMCPFYGHPNPPAPCPCPLKQAWGSLDALVGRLRAAYEENGGKPETNPFGARAVRLYLREVRDLQSKARGVSYEKKKKRKRPTPPPPIPPPQLLSTSVQLPLSSPPPSGKGALPFEL

>SlG7

MMSSSEQKREVGGREGEGSSSMTLTQSDHHHQLSPPPQLSRYESQKRRDWNTFGQYLKNHKPPVPLPQCNYNHVLDFLRYLDQFGKTKVHLNGCVFFGQVEQVGPCTCPLRQAWGSLDALIGRLRAAYEENGGLQETNPFANSAIRIYLREARADEDLKLRINSIAFAILYFYLRIHSISIHISTQYRNQ

>SlG8

NSCHNFWLQKSINHNPFQEFHLKKEKKNNMSNFDRGKELVEGSSPATPSRYESQKRRDWNTFGQYLRNQRPPVSISQCNSNHVLEFLRYLDQFGKTKVHLQGCIFYGQPEPPAPCTCPLRQAWGSLDALIGRLRAAYEENGGSPETNPFASGAIRVYLREVKECQAKARGIPYKKKKKKASESKGDDDCTSSHPFS

>SlG9

MDSFVEVEPSNTTTNNNNITSSSSTSSSRYENQKRRDWNTFGQYLKNHRPPLSLSRCSGAHVLEFLRYLDQFGKTKIHTLICPFYGLPNPPAPCPCPLRQAWGSLDALIGRLRAAYEENGGNPEMNPFGTRAVRLYLREVRDLQSKARGVSYEKKKRKRPSQPSPPPLQSG

>SlG10

MEHNQEVDSPNSVIINHHHHHNHNLDNNSMTMLAGNNNNNNNNSYLASSSSNSPTTLSRYENQKRRDWNTFGQYLRNHRPPLSLTRCSGAHVLEFLRYLDQFGKTKVHTQLCPFFGHPNPPAPCPCPLRQAWGSLDALIGRLRAAYEENGGKPETNPFGARAVRLYLREVRDSQAKARGISYEKKKRKKPNPQHSSSSSLPPPNGNSS

>SlG11

MMSSEKIREVGEGSSSSGGAISIIATPLNNHHRQSSSSSLSTLAPTPASSSAPQLSRYESQKRRDWNTFGQYLKNQRPPISLPQCNYNHVLDFLRYLDQFGKTKVHLHGCLFFGQPEPPGPCTCPLRQAWGSLDALIGRLRAAYEENGGLQETNPFASGAIRVYLREVRDSQAKARGIPYKKKKKKKRPNLQIKASNNNDGATSANFQLQSTT

>SlG12

MSSSDIRGKDLAEGSSRSPGRDQPPSRYESQKRRDWNTFNHYLKNQRPPILLPHCHSNHVLEFLRYLDQFGKTKVHLLGCMFYGQPDPPAPCTCPLRQAWGSLDALIGRLRAAYEENGGSSETNPFASVGIRVYLREVKECQAKARGIAYKKKQKKLANSPSKGDHDDASCPGFLTFS

>SlG13

MSNDQIIIEGEGGGGGGEGSSSRSKTTILIAPSDDHHHHHQLPPVPPQLSRYESQKRRDWNTFGQYLKNQRPPVPLSQCNYNHVLEFLRYLDQFGKTKVHLHGCPFFGQPEPPGPCTCPLRQAWGSLDALIGRLRAAYEENGGLPENNPFASGAIRVYLREVRDFQAKARGICYKKKKKKRKMQNKPTSSNAHEPTTTTFQFQSS

>BdG1

MEKSESEDAPEPKKKAMSRYESQKRRDWATFTRYLAAHRPPLELRRCSGAHVLEFLRHLDRFGKTRVHIPHCPSYGGAALSSSTANTHFHPCQCPLQQAWGSLDALVGRLRAAFADNDNDNNKASINPFAARAVRLYLRDVRDTQSRARGISYAKKKKKKKKKKKKVRTNKTKIGRGKLDDDAAGSGKAAAASSSKTSAENPAASCSGRKTTAMQQAAAPALEPLPPCLPGVPIGCYYDVGLGGLGGGLYLPLLFNAFT

>BdG2

MLQGGVSGPSSAAGSSPISPMSQDASPARPSRYESQKRRDWHTFGQYLRNHRPPLELARCSGAHVLEFLRYLDQFGKTKVHTAGCPFFGHPSPPAPCPCPLRQAWGSLDALVGRLRAAFEEHGGRPEANPFGARAVRLYLREVRDSQAKARGIAYEKKRRKRPSAAAQKPKVKAEPAPEKMNVVAPPPPPPAAQEAADQGHRFFVPHPYHHHHAQFMHHHHGGAHFLLAPVGGGDASSVTASNNDSGGNSGGGDEMAMAMAAAAEAHAAGCMLPLSVFN

>BdG3

MDSSAGAVGGTTTTTTTTTTPSSSSGPGADAHPPAPPPPPQQQLSRYESQKRRDWNTFLQYLRNHRPPLSLARCSGAHVIEFLKYLDQFGKTKVHAPACAHYGDPSPPAPCPCPLRQAWGSLDALIGRLRAAYEESGHAPESNPFAARAVRIYLREVRDAQAKARGIPYEKKKKRKRTQQALPAAQQAAAGEGTSSSAAAGASAATDSASAIAASGGGGGESSGQTGGATAAAAAPAVVPTGQQTGGSGSTTAAPTSAGSR

>BdG4

MPHCETSLLLPPHLNPFHSLSLSHSFSSYLRKLAILSFFVPLPCRQTTRTRQEIRDEKIAKSEREDRAPTMEPAPNTPRGGPASTPEEAAGTSSSSASVEKAEKHEQEAQPREGGGQQQQLAVQAGGHLQPQPLSQQPPPLSAPVPAGLSRYESQKRRDWNTFLQYLSNHKPPLTLARCSGAHVIEFLKYLDQFGKTKVHADGCAYFGQPNPPAPCPCPLRQAWGSLDALIGRLRAAYEESGGRPESNPFAARAVRIYLREVREAQAKARGIPYEKKKRKRGSGSAGPVAASPPVVTAEATSGGGKDDEDQPSRSAEQHQQTTPVVFPTTQTTSSSAGATTATAATKATPTPRGKEPAEGSA

>BdG5

MDSPGAPGPSTAGAGAGSAADGVAAPKPPQLSRYESQKRRDWNTFLQYLRNHRPPLTLARCSGAHVIEFLRYLDQFGKTKVHAAGCAYYGQPNPPGPCPCPLRQAWGSLDALIGRLRAAYEESGGAPESNPFAARAVRIYLREVRDSHAKARGIPYEKKKRKRGTPAEGSSSTAAGGDAGAGGSGSAAAGVSSGSGAAQAPAESGGTAGGTTAPSIS

>BdG6

MDMSGMVAAADSPSGGGGSPLPRPSRYESQKRRDWQTFGQYLRNHRPPLELSRCSGAHVLEFLRYLDQFGKTKVHGPGCPFFGHPSPPGPCPCPLKQAWGSLDALVGRLRAAFEEHGGRAEANPFGARAVRLYLRDVRDSQAKARGIAYEKKRRKRNSNPQKPKPEKKQEKPPNQEAAASEMASAAVGEIRPPELVATPYPPYYLFPHGHLFQGHYLAPASGEGSHAAAAALDGVPGGGAGAGEDIVLVMAAAAAAAEAHAAGCLMPLSVFN

>BdG7

MVNLFVLVNLSLTVPELDRARMDLVPHPDSPHSDNNSGGGGGVAAASSAGASPLSSPSRYESQKRRDWNTFGQYLRNHRPPLSLGRCSGAHVLEFLRYLDQFGKTKVHAQGCPFFGHPNPPAPCPCPLRQAWGSLDALVGRLRAAYEENGGRPENNPFGARAVRLYLREVREHQARARGVSYEKKKRRKNNPAAGPHHGDLNGNSSNGVGHHHHMPPPPPPPPSGAAV

>BdG8

MDHHSPNPESPTAPGSGGSGGGASSSAGGSGVQPQTPSRYEAQKRRDWNTFGQYLRNHRPPLSLSQCSGAHVLEFLRYLDQFGKTKVHTASCPFFGHPNPPAPCPCPLRQAWGSLDALVGRLRAAFEEHGGRPESNPFAARAVRLFLREVREHQARARGVSYEKKKRKKPTAGPGGAEASGSGPQPPPPPPPAGAAC

>BdG9

MDHHHHHHHHHHHMIPGQEPSAPDTNPTPDAFFLGPGGAAIFSGSGGAGSSSSSAAGATSSASPSGSSPSLSRYESQKRRDWNTFGQYLRNHRPPLSLSRCSGAHVLEFLKYMDQFGKTKVHTPMCPFYGHPNPPAPCPCPLRQAWGSLDALIGRLRAAYEENGGTPEMNPFGARAVRLYLREVRETQARARGISYEKKKRKKPSSASGSGSGSAAGGGGGPSSEGSPPPPHPGPPDAPHFIMP

>BdG10

MDHHLKANPDSPLSGGGGSNSSGVGGSSSSSITSSPSSVGNTPQSPSRYEAQKRRDWNTFGQYLRNHRPPLSLAQCSGAHVLEFLRYLDQFGKTKVHASACPFFGHPTPPAPCPCPLRQAWGSLDALVGRLRAAYEENGGSPESNPFAARAVRLYLREVREHQARARGVSYEKKKRKKPQPQLLLQHPGDSSGGGFRQPPPGPPPATGC
