# Supplementary material for: Expression profiling of ALOG family genes during inflorescence development and abiotic stress responses in rice (Oryza sativa L.)
Source: Front Genet. 2024 Apr 8;15:1381690. doi: 10.3389/fgene.2024.1381690 (PMC11033443; doi:10.3389/fgene.2024.1381690)
Supplement: Supplementary file 1 [file DataSheet1.ZIP › ALOG in rice -Figure Legend and Supplementary Files/02-Table S2.docx]

Table S2. The coding sequences (CDS) of rice ALOG members.

>*OsG1*

ATGTCGTCGTCGTCCGCTGCCGCGCTGGGCTCCGACGACGGCTGCTCGCCGGCGGAGCTGCGGCCGAGCCGGTACGAGTCGCAGAAGCGCCGGGACTGGCAGACCTTCACGCAGTACCTCGCCGCGCACCGCCCGCCGCTCGAGCTCCGCCGCTGCAGCGGCGCCCACGTCCTCGAGTTCCTCCGCTACCTCGACCGCTTCGGCAAGACGCGCGTCCACGAGCCGCCGTGCCCGTCGTACGGCGGCCGCTCGCCGTCCGCCGCCGGCCCGGTCGCCGCCGCCGCCGCCGCATGCCAGTGCCCGCTGCGCCAGGCGTGGGGCAGCCTCGACGCGCTCGTCGGCCGCCTCCGCGCCGCCTACGACGAGCGTCACGGCCGCGCCGGGGAGCCCGACGCCGTCGCGGGCGCCGGCGCGGTCGCCACCGACAGTACCTCCTCCTCCTCCGCCGCCGCCGCCAACCCCTTCGCCGCGCGCGCCGTGAGGCTGTACCTGCGCGACGTCCGCGACGCGCAGGCCATGGCGCGCGGCATCTCCTATCACAAGAAGAAGAAGCGCAGGGGCGGCAACATGAACGGCGCCCGCGGCGGCGGCGGCGGCGGCGCGCGCGCGGGCGTGAACGACGGCGATGCGACGGCGCCGCCGGTGGCGGTGACCCCGGGGCTACCTCTGCCGCCGCTGCCACCGTGCCTCAACGGTGTGCCGTTCGAGTACTGCGACTTCGGGAGCGTCCTCGGGGGAGCACATGGCGCCCATGGCGGCCATGGCGGCGGCGGCGGCGGCTTCTACGGCGCCGGCGTCTACTTGCCATTTCTGTACAACACCTTCAGTTAG

>*OsG1L1*

ATGGACATGATCGGCATGGCGTCGCCGGCGGAAAGCCCCGGCGGCGGCGGCACGGCCAGGCCGAGCAGGTACGAGTCGCAGAAGCGGCGGGACTGGCAGACGTTCGGGCAGTATCTGCGCAACCACCGGCCGCCGCTGGAGCTCTCCCGGTGCAGCGGCGCGCACGTGCTGGAGTTCCTCCGGTACCTGGACCAGTTCGGGAAGACGAAGGTGCACGCCCATGGCTGCCCCTTCTTCGGCCACCCCTCGCCGCCGGCGCCGTGCCCCTGCCCGCTCCGCCAGGCGTGGGGCAGCCTCGACGCCCTCGTCGGCCGCCTCCGCGCCGCCTTCGAGGAGCACGGCGGCCGCCCTGAGTCCAACCCCTTCGGCGCCCGCGCCGTCCGCCTCTACCTCCGCGACATCCGCGACACGCAGTCCAAGGCCCGCGGCATCGCCTACGAGAAGAAGCGCCGCAAGCGCGCCGCCGCCTCCCACACCAAGCAGAAGCAGCAGCAGCAGCAGCTGGTGGAACAGGCGGTGGCGCCGCCCGCCGCCGCCGCCGCCGCCGCGGCGCTGCCGGACATGGAGACGACGACGACGACGACCACGGTGCCGCACTTCTTGTTCCCGGCGCACTTCCTCCACGGCCACTACTTCCTGGCACCGGCCGGCGAGCAGCCCGGCGGCGGCGACGTCGCGGCGTCGACGGGCGGCGCTGCCGGTGCTCCCAGCGGCGGCGGCGGGGAGGACCTGGTGCTGGCCATGGCGGCGGCGGCGGCGGCCGCCGAGGCGCACGCCGCCGGCTGCATGATGCCACTGTCGGTGTTCAACTAG

>*OsG1L2*

ATGCAGGGAGGCGGCGGCGGGGATAGCTCCGGCGGCGGCGGCGGCGAGGCGCCGCGGCCGAGCAGGTACGAGTCGCAGAAGCGGCGGGACTGGCACACGTTCGGGCAGTACCTGCGCAACCACCGGCCGCCGCTGGAGCTGTCGCGGTGCAGCGGCGCGCACGTGCTGGAGTTCCTCCGGTACCTGGACCAGTTCGGGAAGACGAAGGTGCACGCCGCGGGGTGCCCCTTCTTCGGCCACCCCTCGCCGCCGGCGCCGTGCCCGTGCCCGCTCCGCCAGGCGTGGGGCAGCCTCGACGCGCTCGTCGGCCGCCTCCGCGCCGCCTTCGAGGAGCACGGCGGCCGCCCCGAGGCCAACCCCTTCGGCGCCCGCGCCGTCCGCCTCTACCTCCGCGAGGTCCGCGACAGCCAGGCCAAGGCCCGCGGCATCGCCTACGAGAAGAAGCGCCGCAAGCGCCCGCCCACCTCCTCGTCTTCGTCTCAGGCCGCCGCCGCCGCCGCCGCCGCCACCTCCCCGGCGAGCCCAGCCGCTAGCCCGACGCCGCCACCGCCACCGCCGACGGAGAGATCAGCCGACGTGCGGCCGATGCCACCGGAGGGCCACTTCTTCATCCCGCACCCACACTTCATGCACGGACACTTCCTCGTACCGGGCGGCGACGCCGACCACCACCACCAGGTCTCCAACGCCGGCAATGGCGGCAACACCAACACCAACACCAACACCAACACCGGCGGCGGCGGCGGCAACGGCGACGAGATGGCGGTGGCGATGGCGGCGGTGGCGGAGGCGCACGCGGCGGGGTGCATGCTGCCGCTGTCCGTGTTTAACTAG

>*OsG1L3*

ATGGAGCTGTCGCCGCCGAACCACGAGAGCAGTCCACCGACGGCGGGCGGCGGCGGCGGAGGAGGAGGTGACGGCGCCGGCGGATCGAGCAGCGCGGGCGCGTCGTCGTCGGCGGGAGGCGGCGCCGCCACGCCGCAGACGCCGAGCCGGTACGAGGCGCAGAAGCGGCGGGACTGGAACACGTTCGGGCAGTACCTGCGGAACCACCGGCCGCCGCTGGGGCTCGCGCAGTGCAGCGGCGCGCACGTGCTGGAGTTCCTCCGGTATCTGGACCAGTTCGGGAAGACGAAGGTGCACACGGCGGCGTGCCCCTTCTTCGGCCACCCGAACCCGCCGGCGCCGTGCCCCTGCCCGCTGCGCCAGGCGTGGGGGAGCCTCGACGCGCTGGTCGGCCGCCTCCGCGCCGCGTTCGAGGAGAACGGCGGGCGGCCGGAGTCGAACCCGTTCGCGGTGCGCGCGGTGAGGCTCTACCTCCGCGAGGTCCGCGAGCACCAGGCCCGCGCCAGGGGGGTCAGCTACGAGAAGAAGAAGCGGAAGAAGCCGCAGCCCGCCGACACCAGCGGCGGCGGCGGACACCCGCACCCGCCGCCGCCTCCGCCGCCGCCGCCGTCCGCCGGCGCGGCCTGCTGA

>*OsG1L4*

ATGGACCTGTCGCCGAACCCCGACAGCCCTCCGTCGGGAGGGGGCAACGGCGGCGGCGGTGGGTCGAGCAGCAGCAACTCGTCGCCGTCCATGGGCGCGGGGGCGCCGCAGTCGCCGAGCCGGTACGAGGCGCAGAAGCGGCGCGACTGGAACACGTTCGGGCAGTACCTGCGGAACCACCGGCCGCCGCTGAGCCTCGCGCAGTGCAGCGGCGCGCACGTCCTGGAGTTCCTCCGCTACCTGGACCAGTTCGGCAAGACCAAGGTGCACACCGCGGCGTGCCCCTTCTTCGGCCACCCGAGCCCGCCGGCGCCGTGCCCCTGCCCGCTCCGCCAGGCCTGGGGCAGCCTCGACGCCCTCGTCGGCCGCCTCCGCGCCGCCTTCGAGGAGAACGGCGGCCGCCCGGAGTCCAACCCCTTCGCCGCGCGCGCCGTCCGCCTCTACCTCCGCGAGGTCCGCGAGCACCAGGCGCGCGCCCGCGGCGTCAGCTACGAGAAGAAGAAGCGCAAGAAGCCGCAGCAGCAGCAGCTGCAGGGCGGCGACAGCAGTGGCCTTCACGGCCACCAGCATCACCCGCCGCCTCCACCGCCTGCCGGCGCCGCCTGCTGA

>*OsG1L5*

ATGGAGTTCGTGGCGCACGCGGCGGCGCCGGACAGCCCGCACTCGGACAGCGGCGGAGGAGGAGGGGGAATGGCGACGGGGGCGACGTCGGCGTCGGCGGCGGGGGCGTCGCCGAGCAGGTACGAGTCGCAGAAGCGGCGGGACTGGAACACGTTCGGGCAGTACCTCCGCAACCACCGGCCGCCGCTGTCGCTGGCGCGGTGCAGCGGCGCGCACGTCCTGGAGTTCCTCCGCTACCTGGACCAGTTCGGCAAGACCAAGGTGCACGCGCCGGCGTGCCCCTTCTTCGGCCACCCGGCGCCGCCGGCGCCGTGCCCGTGCCCGCTTCGCCAGGCGTGGGGCAGCCTCGACGCCCTCGTCGGCCGCCTCCGCGCCGCCTACGAGGAGAACGGCGGCCGCCCCGAGAACAACCCCTTCGGCGCCCGCGCCGTCCGCCTCTACCTCCGCGAGGTCCGCGAGCACCAGGCGCGCGCACGCGGCGTCAGCTACGAGAAGAAGAAGCGCAAGAAGCCACCCCACCCCTCCTCCGCCGCCGCCGCGCACGACGACGCCGCCAACGGCGCCCTCCACCACCACCACCACATGCCGCCGCCTCCTCCCGGCGCCGCCGCCTGA

>*OsG1L6*

ATGGATCGTCACCATCACCACCACCACCACCACCACCATCACATGATGTCGGGCGGCGGGCAAGACCCGGCGGCGGGGGACGGCGGCGCCGGCGGCGCCACGCAGGACAGCTTCTTCCTCGGCCCGGCCGCGGCCGCCATGTTCTCCGGCGCCGGGTCGTCGTCGTCGGGCGCGGGGACGTCGGCGGGAGGCGGCGGCGGCGGGCCATCGCCGTCCAGCTCGTCGCCGTCGCTGAGCCGGTACGAGTCGCAGAAGCGGCGGGACTGGAACACGTTCGGGCAGTACCTGCGGAACCACCGGCCGCCGCTGTCCCTGTCGCGGTGCAGCGGCGCGCACGTGCTGGAGTTCCTCAAGTACATGGACCAGTTCGGGAAGACGAAGGTGCACACGCCGGTGTGCCCCTTCTACGGCCACCCCAACCCGCCGGCGCCATGCCCGTGCCCGCTCCGCCAAGCCTGGGGCTCCCTCGACGCGCTCATCGGCCGCCTCCGCGCCGCCTACGAGGAGAACGGCGGCACGCCGGAGATGAACCCCTTCGGCGCCCGCGCCGTCCGCCTCTACCTGCGCGAGGTGCGCGAGACGCAGGCCAGGGCGAGGGGGATCAGCTACGAGAAGAAGAAGCGCAAGAAGCCCTCGTCCGCCGGCGCCGGAGCCGGGCCCTCGTCCGAGGGGAGCCCGCCGCCACCCGGCGGCTCGGCCAGCGGCGGCGGCGACACGTCGGCGTCGCCGCAGTTCATCATCCCGTGA

>*OsG1L7*

ATGGATCCGTCTGGCCCCGGTCCGTCCTCTGCGGCGGCTGGCGGCGCGCCGGCCGTGGCGGCGGCGCCGCAGCCGCCGGCGCAGCTGAGCAGGTACGAGTCGCAGAAGCGGAGGGACTGGAACACGTTCCTGCAGTACCTGCGGAACCACCGGCCGCCGCTGACGCTGGCGCGGTGCAGCGGCGCGCACGTGATCGAGTTCCTGAGGTACCTGGACCAGTTCGGGAAGACCAAGGTGCACGCGTCGGGGTGCGCCTTCTACGGCCAGCCCAGCCCGCCGGGGCCGTGCCCGTGCCCGCTGCGTCAGGCGTGGGGATCCCTCGACGCGCTCATCGGCCGCCTCCGCGCCGCGTACGAGGAGAGCGGCGGCACGCCCGAGTCCAACCCGTTCGCCGCGCGCGCCGTCCGGATCTACCTCCGCGAGGTGCGGGACTCGCAGGCCAAGGCGCGCGGCATCCCGTACGAGAAGAAGAAGCGCAAGCGCTCGCAGGCGGCGCAGCCCGCCGGCGTCGAGCCGTCCGGCTCGTCTTCTGCTGCAGCTGCAGCTGCCGGTGGTGGAGACGCGGGCAGCGGTGGCGGTGCAGCTGCTACTACCACAGCTCAACCTGGAGGGAGTGGCACTGCACCAAGCGCCTCCTGA

>*OsG1L8*

ATGGAGGGAGGAGGAGGTGGGGCGGACGGGCAGGCGCAGCCGGTGGCGCAGGCGCCGCCGGCGATGCAGCCGATGCAGCAGCTGAGCAGGTACGAGTCGCAGAAGAGGAGGGACTGGAACACGTTCCTGCAGTACCTGAAGAACCACCGGCCGCCGCTGACGCTGGCGAGGTGCAGCGGCGCGCACGTCATCGAGTTCCTCAAGTACCTGGATCAGTTCGGGAAGACGAAGGTGCACGCGTCGGGGTGCGCCTACTACGGCCAGCCGAGCCCGCCGGCGCCGTGCCCGTGCCCGCTGCGCCAGGCGTGGGGGTCCCTCGACGCGCTCATCGGGCGCCTCCGCGCCGCCTACGAGGAGAGCGGCCACGCGCCGGAGTCCAACCCCTTCGCCGCGCGCGCCGTCCGGATCTACCTCCGCGAGGTCCGCGACGCGCAGGCCAAGGCCCGCGGGATACCATACGAGAAGAAGAAGCGCAAGCGCACGCAGCAGCAGCAGCCTCCCCCACCGCCGCCGCCGCCGCCCCAGCACCAGCCGGGCGCCGCCGCCGGGGAGGCGTCGAGCTCGTCGTCTGCTGCCGCCGCCGCCGTCGCAGCAGAAGGCAGTGGCAGCTCCGCCGCCGCCGCCGCCGCCACTAGCCAGACAGGAGGAGGAGGAGGAGGAAGCACCACCACCACCACCGCTTCTGCTGCTGCACCGACCACCGCCACCCGAGTATAG

>*OsG1L9*

ATGGAGCCTTCCCCCGACGCGCCACGCGCCGGGGCAGCGGAAGAGCAGCCTGGTCCGTCCTCCTCGGCGTCGGCGCCGGCGCCCGCGGCGTCGTCGAACGAAGAAGAAGGGAGACATCAGTCGCAGGCGCAGCAGCAGGTGCAAGAAGCGCAGCCGCAGCCGCTGGCGCAGCAGGCGCCGGCGGCGGCGGGGCTGAGCAGGTACGAGTCGCAGAAGCGGCGGGACTGGAACACGTTCCTGCAGTACCTGCGGAACCACAAGCCGCCGCTGACGCTGCCGCGGTGCAGCGGCGCGCACGTCATCGAGTTCCTCAAGTACCTGGACCAGTTCGGGAAGACCAAGGTGCACGCCGACGGGTGCGCCTACTTCGGCGAGCCCAACCCGCCGGCGCCGTGCGCCTGCCCGCTCCGCCAGGCGTGGGGCAGCCTCGACGCGCTCATCGGCCGCCTCCGCGCCGCCTACGAGGAGTCCGGCGGCCGCCCGGAGTCCAACCCCTTCGCCGCCCGCGCCGTCCGCATCTACCTCCGCGAGGTCCGCGAGGCGCAGGCCAAGGCGCGTGGGATCCCCTACGAGAAGAAGCGCAAGCGTGGCGCCGCCGCCGCCGCCGCCGCCCCTCCCGTCGTCGTCGCGCCGCCCCCCGTCGTCACCGCGCCCGACGACGCCACCGGGACGTCGGGAGGCGCCGGCGAGGACGACGACGACGACGAGGCCACCCACTCCGGCGAGCAGCAGGACACCACGCCGGCGGCCTCTCCTACTACTCCTCCGGCAACAAGTGTCGGTACTACCACCGCGGCGGCGACTGCGGCGGCGGCGAAGGGAAGTGCGGCGAAAGGATCGGCGACGAGTTCGTGA

>*OsG1L10*

ATGGCAAAACATACTCGGAAATCATTTATATCATTTGAGCCCGATTACGCTCGCTTCATGCATCATCACATGAAGAATGCTTCATGTACATCTTTTCATAGCCTTACATACACTACCAGGATGGGAGACACTCCCGGGTATGAGCAGAAAGTTTATGTAGTGTGCTTCTATCACTCCGTAAATTACCGGGTGTTTCAGGGGAACACTCTGCAACAACTTCTGTTACGGAGTGTTCATTTAGAACACTGGGGAACACCAGGATATTGGAGTATTACACTAGCGAATATGGCGAGGACAGCGGCCGGCAGAGTTGAGCGTGGCGGTGGCCGCGGTGGACGAGCGTGTGGCCGCCGGAGCCACCCCTCATCGCCGGCGCCATGGCCGTGCCCGCTCCGCCAGGCGTGGGGCAGCCTCGACGTGCTCGTCGGCCGCCTCCGCACCGCCTTCGACGAGCACGGAGGCCACCCCGAGGCCAACCCGTTCGGCGCCCGCGTTGTCCGCCTCTACCTCCGCGAGGTCTGTGACAGCCAGGCCAAGGTGCGCGGCATCGCCTACGAGAAGAAGCGTCGGAAGCGCCCGCCCACCTCGTCTTCTCACTCTCAGGACGGCACAGCCGCCACTTGCCCGGCGAGCCCAGCCGCTAGCCCGACGCCGCTACCGCCGCCGCCGGAGAGATCAGCTGACATGGGAGCTTGCGTCGCCATCGTCGTCGCCGTGGGGTGCACGCCACTCTCGCTAGCCGCCCGCCGCGGCTGCTCCTATTGCGCGCTCGCTCGCCGCCGCTAG

>*OsG1L11*

ATGCCCTTGGGCCCACATATCAGCTCTATCTCCTGTCTCTCTCCTCATCTCTCTTTTCTCTCTCAGATGGCGAGGACGGCGGCCGGCAGAGTTGAGCGGGGCGGTGGCCGCGGTGGACGAGCGTGTGGCCACCGGAGCCACCCCTCGCCGCCAACGCTGTGCCCGTGCCCGCTCCGCCAGGCGTGGGGCAGCCTCGACACGCTCGTCGGCCGCCTCTGCACCGCCTTCGACGAGCATGGAGGCCACCCCGAGGCCAACCCGTTCGGTGCCCGCGTTGTCCGCCTCTACGTCCGCGACAGCCAGGCCAAGGTGCGCGGCATCGCCTACGAGAAGAAGCGTCGGAAGCGCCCTCCCACCTCGTTTTCTCACTCTCAGGCCGCCGCCGCCGCCACTTGCCCGGCGAGCCCAGCCGCTAGCCCGACGCCGGAGAGATCAGCCGACATGGGAGCTTGCGTCGCCATCGCTGTCGCCGTGGGGTGCACGCCACTCTCGCTAGCCGCCCGCCGCGGCTGCTCCTATTGCGCGCTCGCTTACCGCCGCTAG

>*OsG1L12*

ATGTCAGTTCTATCTCCTATCTCTCTCCTCATCTCTCTCTTTTCTCTCTCTTTTCTCTCTCAGATGGCGAGGACAGCGGCCGGCGGGGTGGAGCGGGGCAGCGGCAGCGGTGGACGAGTGCGCAGCCGGCGGAGCCACCCCTCGCCGCTGGCGCCGTGCCCATGCCCGCTCCGCCAGGCGTGGGGCAGCCTCGACGTGCTCGTCGGCCACCTCCGCGCCGCCTTCGAGGAGCACGGCGGCCACCCCGAGGCCAACCCATTCAGCGCCCGCGCCGTCCGCCTCTACCTCCATGAGGTCCGCGACAGCTAG

>*OsG1L13*

ATGGCGAGGACGGTGGCTGGCGGAGTGGAGCGGGGCGGCGGCGGCGGTGGACGAGCGCGCGGCCGGCGGAGCCACCCCTCGCTACCGGTGCCGTGCCCGTGCCTTCTCCGCCAGGCGTGGGGCAGCCTCAACGCGCTCGTCGGCCGCTTCCGCGCCGCCTTTGAGGAGCATGGCGGCCAACCCGAGGCCAACCCGTTCGGCGCCCGCGCCGTCCGCCTCTACCTCCACGAGGTCTACGACTGCCAGGCCAAGGCGCGCGGCATCGCCTACGAGAAGAAGCGTTGGAAACGCCCGCCCACCTCGTCTTCTCACTCTCAGGCCGCCGCCGCCGCTACCTCCTCGGCGAGCCAGCCGCTAGCCCGCTGCCGCCACCGCCGTTGCCGGAGAGATCAGCCGACGTGGTTGACTGTGCCTGATTCCTTAATGGTGGTTTTATGTGCACAAGTGCAAATTAAGAGCAAAGCCGAGCGTAAAAGGCTGTGGATGCTGCCATCTGATTCACCTTCAACTGGAACCCATCTGACCTTAAAGGACAGCTCAGAAATTTCAGAGAATACAATTAGTTGGTAA
