# Supplementary material for: Expression profiling of ALOG family genes during inflorescence development and abiotic stress responses in rice (Oryza sativa L.)
Source: Front Genet. 2024 Apr 8;15:1381690. doi: 10.3389/fgene.2024.1381690 (PMC11033443; doi:10.3389/fgene.2024.1381690)
Supplement: Supplementary file 1 [file DataSheet1.ZIP › ALOG in rice -Figure Legend and Supplementary Files/07-Table S7.docx]

Table S7. Rice Functional Genomic Express Database of Rice ALOG mumbers from GSE data.

| Gene name | YS | YR | YL | ML | SAM | P1 | P2 | P3 | P4 | P5 | P6 | Sti | OV | S1 | S2 | S3 | S4 | S5 |
| --- | --- | --- | --- | --- | --- | --- | --- | --- | --- | --- | --- | --- | --- | --- | --- | --- | --- | --- |
| *OsG1* | 81.8 | 68.9 | 72.4 | 70.2 | 178.4 | 461.6 | 320.9 | 235.9 | 172.1 | 115 | 66 | 34.2 | 76.3 | 68.5 | 98.1 | 69.5 | 117.8 | 160.6 |
| *OsG1L1* | 211.9 | 389.3 | 53.5 | 38.9 | 2108 | 612.1 | 475 | 288.1 | 321.4 | 358.8 | 239.2 | 32.3 | 400.5 | 593.3 | 331.8 | 285.2 | 348.6 | 555.5 |
| *OsG1L2* | 1433.6 | 2873 | 118.7 | 57.8 | 4339 | 1795.4 | 2323.1 | 1464.8 | 1127.5 | 716.6 | 613.5 | 46.2 | 774.5 | 869.2 | 770.2 | 1243.6 | 2905 | 4166.6 |
| *OsG1L4* | 196.3 | 30.4 | 35.6 | 38.3 | 6614.1 | 28.1 | 36.7 | 22.5 | 17.7 | 30.3 | 38 | 20.6 | 35 | 39.9 | 22 | 19.6 | 55.6 | 6.4 |
| *OsG1L5* | 55.2 | 63.1 | 77.4 | 44.6 | 55 | 46.9 | 55.3 | 41.8 | 42.4 | 33.8 | 35.7 | 35.6 | 17.8 | 31.3 | 55.5 | 44.6 | 19.9 | 16.3 |
| *OsG1L6* | 91.2 | 51.5 | 72.1 | 60.8 | 457.5 | 153.1 | 105.7 | 154.4 | 102.1 | 112.4 | 49.7 | 36.8 | 38.2 | 71.4 | 46.5 | 43.6 | 26.4 | 90.3 |
| *OsG1L7* | 21.6 | 26.4 | 36.7 | 37 | 36.7 | 28.3 | 33 | 22 | 21.9 | 23.7 | 25.8 | 24.9 | 31.6 | 25.9 | 30.1 | 33.9 | 39.7 | 44.2 |
| *OsG1L8* | 112.8 | 329.1 | 66.5 | 57.6 | 217.3 | 51.6 | 41.6 | 60.6 | 107.4 | 26.6 | 111.7 | 25 | 256.6 | 136.7 | 128.9 | 147.2 | 180.5 | 188.1 |
| *OsG1L9* | 193.3 | 726.1 | 59.8 | 110.5 | 344.4 | 77.5 | 44.2 | 43.7 | 44.1 | 74.8 | 90.5 | 21.2 | 27.4 | 86.7 | 444.1 | 804.9 | 711.9 | 95.1 |
| *OsG1L10* | 19.7 | 35.5 | 38.4 | 40.2 | 30.9 | 29.2 | 29.6 | 37.4 | 28.1 | 24.6 | 39.8 | 16.8 | 11.7 | 26.2 | 30.4 | 24.7 | 19.9 | 30.1 |
| *OsG1L11* | 189.6 | 129.2 | 120.7 | 148.3 | 155.1 | 138.1 | 107.9 | 82.2 | 139.7 | 130.3 | 115.9 | 72.4 | 84.2 | 87.4 | 115 | 83.1 | 219.4 | 99.3 |
| *OsG1L12* | 19.2 | 33.3 | 56.9 | 33.8 | 49.7 | 41.2 | 51.7 | 24.5 | 28.2 | 24.3 | 30.9 | 16.1 | 11.6 | 17.6 | 19.4 | 27.1 | 12.9 | 26.2 |
| *OsG1L13* | 43.5 | 52.2 | 60.9 | 51.2 | 49 | 43.4 | 48.9 | 47.1 | 49 | 36.6 | 51.6 | 36.1 | 16.6 | 45.8 | 49.5 | 45.6 | 41.9 | 28.2 |

The numbers in the table represent the relative expression level of the related genes. YS, 7-day-old seedlings; YR, roots from 7-day-old seedlings; YL, leaves from 7-day-old seedlings; ML, mature leaf; SAM, shoot apical meristem; different stages of panicle development: P1, 0-3 cm; P2, 3-5 cm; P3, 5-10 cm; P4, 10-15 cm; P5, 15-22 cm; P6, 22-30 cm; Sti, stigma of mature pistil; OV, mature ovary; different stages of seed development: S1, 0-2 dap (day after pollination); S2, 3-4 dap; S3, 5-10 dap; S4, 11-20 dap; S5, 21-29 dap.
