# Supplementary material for: Expression profiling of ALOG family genes during inflorescence development and abiotic stress responses in rice (Oryza sativa L.)
Source: Front Genet. 2024 Apr 8;15:1381690. doi: 10.3389/fgene.2024.1381690 (PMC11033443; doi:10.3389/fgene.2024.1381690)
Supplement: Supplementary file 1 [file DataSheet1.ZIP › ALOG in rice -Figure Legend and Supplementary Files/04-Table S4.doc]

Table S4. Primers used in subcellular localizations of rice ALOG proteins.

| **Proteins** | **Primers used in qRT-PCR (5′→3′)** | |
| --- | --- | --- |
| OsG1L3 | Forwards： | AAGTCCGGAGCTAGCTCTAGatggagctgtcgccgccg |
|  | Reverse： | AGCGGCCGCTGTACAGGATCgcaggccgcgccggcggac |
| OsG1L7 | Forwards： | AAGTCCGGAGCTAGCTCTAGatggatccgtctggccccg |
|  | Reverse： | AGCGGCCGCTGTACAGGATCggaggcgcttggtgcagtg |
