# Supplementary material for: Expression profiling of ALOG family genes during inflorescence development and abiotic stress responses in rice (Oryza sativa L.)
Source: Front Genet. 2024 Apr 8;15:1381690. doi: 10.3389/fgene.2024.1381690 (PMC11033443; doi:10.3389/fgene.2024.1381690)
Supplement: Supplementary file 1 [file DataSheet1.ZIP › ALOG in rice -Figure Legend and Supplementary Files/05-Table S5.doc]

Table S5. Primers used in qRT-PCR of rice ALOG genes.

| **Genes** | **Primers used in qRT-PCR(5′→3′)** | |
| --- | --- | --- |
| *OsG1* | Forward： | GAGCCGGTACGAGTCGCAGAA |
|  | Reverse： | CGGTCGAGGTAGCGGAGGAAC |
| *OsG1L1* | Forward： | CTGGTGGAACAGGCGGTGGC |
|  | Reverse： | AAGTGCGCCGGGAACAAGAA |
| *OsG1L2* | Forward： | CCACCCTACTCCTTCTAGTACCTC |
|  | Reverse： | CAGCAAACCTTTCTCCCTCCT |
| *OsG1L3* | Forward： | CAGACGCCGAGCCGGTACGA |
|  | Reverse： | TTCGGGTGGCCGAAGAAGGG |
| *OsG1L4* | Forward： | AGCAACTCGTCGCCGTCCAT |
|  | Reverse： | GGCCGGTGGTTCCGCAGGTA |
| *OsG1L5* | Forward： | GTCGCCGAGCAGGTACGAGT |
|  | Reverse： | CCTTGGTCTTGCCGAACTGG |
| *OsG1L6* | Forwards： | CTGCTGCCTTGCTTGCTGTCA |
|  | Reverse： | CATGCACGAGCACGAGACGAG |
| *OsG1L7* | Forward： | GAAGCGGAGGGACTGGAACA |
|  | Reverse： | GCTGGGCTGGCCGTAGAAGG |
| *OsG1L8* | Forward： | CGCGGGATACCATACGAGAA |
|  | Reverse： | CGGAGCTGCCACTGCCTTCT |
| *OsG1L9* | Forwards： | CAGCCTGGTCCGTCCTCCTC |
|  | Reverse： | TCCCGCCGCTTCTGCGACTC |
| *OsG1L11* | Forward： | CCTCCCACCTCGTTTTCTCA |
|  | Reverse： | TGGCGACGCAAGCTCCCATG |
| *OsG1L12* | Forward： | AGCGGCAGCGGTGGACGAGT |
|  | Reverse： | ATGGAGGTAGAGGCGGACGG |
| *OsG1L13* | Forward： | GACTGTGCCTGATTCCTTAATGGT |
|  | Reverse： | CAGATGGGTTCCAGTTGAAGGT |
