# Supplementary material for: Expression profiling of ALOG family genes during inflorescence development and abiotic stress responses in rice (Oryza sativa L.)
Source: Front Genet. 2024 Apr 8;15:1381690. doi: 10.3389/fgene.2024.1381690 (PMC11033443; doi:10.3389/fgene.2024.1381690)
Supplement: Supplementary file 1 [file DataSheet1.ZIP › ALOG in rice -Figure Legend and Supplementary Files/06-Table S6.doc]

Table S6. Physical and chemical properties of rice ALOG numbers.

| Gene name | Gene ID | Chromosomal location | E-value | Number of amino acids | Molecular weight | Predicted pI | Predicted subcellular localization |
| --- | --- | --- | --- | --- | --- | --- | --- |
| *OsG1* | *Os07g04670* | 2068174 - 2067344 | 1.5e-47 | 276 | 28496.8691 | 9.54 | Nucleus |
| *OsG1L1* | *Os02g07030* | 3594309 - 3596594 | 1.1e-65 | 270 | 28628.3809 | 9.48 | Nucleus |
| *OsG1L2* | *Os06g46030* | 27906635 - 27904549 | 3e-66 | 277 | 29165.4805 | 9.56 | Nucleus |
| *OsG1L3* | *Os02g41460* | 24832108 - 24830541 | 1.4e-65 | 209 | 21808.3203 | 10.14 | Nucleus |
| *OsG1L4* | *Os04g43580* | 25791567 - 25790014 | 1.5e-66 | 202 | 21673.1699 | 10.30 | Nucleus |
| *OsG1L5* | *Os10g33780* | 17889724 - 17888297 | 2.2e-66 | 204 | 21835.4609 | 10.21 | Nucleus |
| *OsG1L6* | *Os02g56610* | 34689723 - 34687624 | 3.3e-66 | 248 | 25885.6602 | 9.97 | Nucleus |
| *OsG1L7* | *Os01g61310* | 35474333 - 35473695 | 4e-66 | 212 | 21743.25 | 10.27 | Nucleus |
| *OsG1L8* | *Os05g39500* | 23178205 - 23178921 | 1.4e-64 | 238 | 24725.6309 | 10.15 | Nucleus |
| *OsG1L9* | *Os05g28040* | 16364011 - 16362685 | 7.5e-65 | 284 | 29440.3691 | 6.36 | Nucleus |
| *OsG1L10* | *Os01g54180* | 31163139 - 31165152 | 3.1e-33 | 261 | 28749.82 | 10.40 | Nucleus |
| *OsG1L11* | *Os05g27120* | 15779933 - 15779391 | 2.4e-35 | 180 | 18931.7402 | 10.44 | Chloroplast |
| *OsG1L12* | *Os08g09660* | 5580577 - 5580885 | 6.6e-33 | 102 | 10931.5 | 10.76 | Extracellular |
| *OsG1L13* | *Os08g14970* | 9031356 - 9030093 | 9e-30 | 189 | 20726.6191 | 11.12 | Chloroplast |
