# Supplementary material for: Expression profiling of ALOG family genes during inflorescence development and abiotic stress responses in rice (Oryza sativa L.)
Source: Front Genet. 2024 Apr 8;15:1381690. doi: 10.3389/fgene.2024.1381690 (PMC11033443; doi:10.3389/fgene.2024.1381690)
Supplement: Supplementary file 1 [file DataSheet1.ZIP › ALOG in rice -Figure Legend and Supplementary Files/Figure 1-7 legend.doc]

Figure 1. Multiple sequence alignment and 3D analysis of ALOG proteins. (**A**) Multiple sequence alignment of the ALOG proteins from rice and *Arabidopsis thaliana*. Multiple alignments of the highly conserved ALOG domains (134 aa) of ALOG proteins. The conserved ALOG domain included Helix-1/2/3/4 and a zinc ribbon insert structure. (**B**) Prediction of three-dimensional structure of 14 ALOG proteins from rice. Helix-1, Helix-2, Helix-3 and Helix-4 were marked in red, and the zinc ribbon insert was marked in yellow.

Figure 2. Phylogenetic tree of ALOG domain proteins in five different plant species. On the basis of the full-length sequences of ALOG proteins, a total of 59 proteins were used to construct the phylogenetic tree. Species abbreviations were as follows: Os, *Oryza sativa*; At: *Arabidopsis thaliana*; Bd: *Brachypodium distachyon*; Sl: *Solanum lycopersicum*; Sb: *Sorghum bicolor*. Clades A-I were indicated in different colored circles.

Figure 3. Collinearity analysis of ALOG genes. (**A**) Collinearity relationship between ALOG genes from rice with *Arabidopsis* and *Brachypodium distachyon*. The chromosomes of rice, *Arabidopsis* and *Brachypodium distachyon* were marked with different colors. (**B**) Collinearity relationship of ALOG genes from rice. The collinear relationship between the ALOG family members of different species were connected by red lines.

Figure 4. The subcellular localization of OsG1L3 and OsG1L7. (**A**) The signal of 35S::OsG1L3-GFP fusion protein; (**B**) The signal of 35S::OsG1L7-GFP fusion protein. The 35S::OsG1L3/7-GFP and control vector (35S::GFP) were transiently expressed in rice protoplasts. Confocal microscopy images were visualized after 16 h transformation. Scale bars = 10 μm.

Figure 5. Expression profiles of ALOG genes in various tissues. (**A**) The expression of microarray data of ALOG genes in various organs at different stages. The heat map representing hierarchical cluster was generated by using the average log2 expression values of ALOG genes in various organs, including YS, 7-day-old seedlings; YR, roots from 7-day-old seedlings; YL, leaves from 7-day-old seedlings; ML, mature leaf; SAM, shoot apical meristem; different stages of panicle development: P1, 0-3 cm; P2, 3-5 cm; P3, 5-10 cm; P4, 10-15 cm; P5, 15-22 cm; P6, 22-30 cm; Sti, stigma of mature pistil; OV, mature ovary; different stages of seed development: S1, 0-2 dap (day after pollination); S2, 3-4 dap; S3, 5-10 dap; S4, 11-20 dap; S5, 21-29 dap. The differential expression of ALOG genes in various organs were consistent with the results of qRT-PCR analysis, which were represented by the asterisk (*) on the right. The color scale (representing the average log signal value) was displayed on the right side. (**B**) The expression of ALOG genes (marked by asterisks in A) in different organs using qRT-PCR, which were consistent with microarray data using qRT PCR. The error bar represented the standard deviation of three independent biological replicates (n = 3). Different letters (a, b, c, d) denoted significant differences at *P* < 0.05 according to ANOVA in combination with Duncan’s multiple range test.

Figure 6. The expression profiles of ALOG genes in inflorescence. (**A**) The ALOG genes mainly expressed at P1 stage of inflorescence. (**B**) The ALOG genes predominately expressed at P4 stage of inflorescence. The stages of P1-P6 were represented by the spikelet length of 0-1, 1-3, 3-5, 5-10, 10-15, 15-22 cm. The y-axis was used to detect the relative expression level of the genes at different stages of inflorescence using qRT-PCR. The error bar represented the standard deviation of three independent biological replicates (n = 3). Different letters (a, b, c, d) denoted significant differences at *P* < 0.05 according to ANOVA in combination with Duncan’s multiple range test.

Figure 7. Prediction of cis-acting elements related to hormone and abiotic stress responses in the promoter region and expression analysis of ALOG genes in rice. (**A**) The number of *cis*-acting elements detected in the putative promoter region (the sequence was retrieved from the 3 kb region upstream of the start codon).The *cis*-acting elements were divided into 10 categories. (**B**) The number, type and location of stress-related and hormone-responsive elements in the putative promoter regions were speculated by the ALOG genes in Fig. 7 A. TGA-element and TGA-box, Auxin responsive elements; ABRE, ABA responsive elements; CGTCA-motif and TGACG-motif, MeJA responsive elements; MBS, drought responsive elements; TATC-box and P-box, GA responsive elements; TCA-element, SA responsive elements; TC-rich repeats, defense and stress responsive elements. Different types of elements were represented by different colors. (**C**) The expression analysis of ALOG genes under ABA treatment. (**D**) The expression analysis of ALOG genes under drought treatment. The y axis was the relative expression level of the genes compared to Actin under different treatments using qRT-PCR. The expression level was based on the relative expression level of Actin at 0 h as a reference. The error bar represented the standard deviation of three independent biological replicates (n = 3). Different letters (a, b, c, d) denoted significant differences at *P* < 0.05 according to ANOVA in combination with Duncan’s multiple range test.
