# Supplementary material for: Expression profiling of ALOG family genes during inflorescence development and abiotic stress responses in rice (Oryza sativa L.)
Source: Front Genet. 2024 Apr 8;15:1381690. doi: 10.3389/fgene.2024.1381690 (PMC11033443; doi:10.3389/fgene.2024.1381690)
Supplement: Supplementary file 1 [file DataSheet1.ZIP › ALOG in rice -Figure Legend and Supplementary Files/Figure S1-S5 Legend.doc]

Figure S1. Structures analysis of rice ALOG members. (**A**) The phylogenetic trees of rice ALOG family. (**B**) Gene structures of rice ALOG members. UTR, untranslated region; exon, expressed region. (**C**) The motifs of rice ALOG proteins. The order of the motifs corresponds to their position within individual protein sequences.

Figure S2. The expression of ALOG genes in different organs. The error bar represented the standard deviation of three independent biological replicates (n = 3). Different letters (a, b, c, d) denoted significant differences at *P* < 0.05 according to ANOVA in combination with Duncan’s multiple range test.

Figure S3. The expression profiles of ALOG genes in inflorescence. The stages of P1-P6 were represented by the spikelet length of 0-1, 1-3, 3-5, 5-10, 10-15, 15-22 cm. The y-axis was used to detect the relative expression level of the genes at different stages of inflorescence using qRT-PCR. The error bar represented the standard deviation of three independent biological replicates (n = 3). Different letters (a, b, c, d) denoted significant differences at *P* < 0.05 according to ANOVA in combination with Duncan’s multiple range test.

Figure S4. Expression analysis of ALOG genes under ABA treatment. The expression level was based on the relative expression level of Actin at 0 h as a reference. The error bar represented the standard deviation of three independent biological replicates (n = 3). Different letters (a, b, c, d) denoted significant differences at *P* < 0.05 according to ANOVA in combination with Duncan’s multiple range test.

Figure S5. Expression analysis of ALOG genes under drought treatment. The expression level was based on the relative expression level of Actin at 0 h as a reference. The error bar represented the standard deviation of three independent biological replicates (n = 3). Different letters (a, b, c, d) denoted significant differences at *P* < 0.05 according to ANOVA in combination with Duncan’s multiple range test.
